# Cellular SLC35B4 promotes internalization during influenza A virus entry

**DOI:** 10.1128/mbio.00194-25

**Published:** 2025-03-25

**Authors:** Guangwen Wang, Li Jiang, Ya Yan, Fandi Kong, Qibing Li, Jie Zhang, Shuangshuang Hou, Bo Wang, Xiurong Wang, Huihui Kong, Guohua Deng, Jianzhong Shi, Guobin Tian, Xianying Zeng, Hualan Chen, Chengjun Li

**Affiliations:** 1 State Key Laboratory for Animal Disease Control and Prevention, Harbin Veterinary Research Institute, Chinese Academy of Agricultural Sciences 687216 , Harbin, Heilongjiang, China; Washington University in St. Louis, St. Louis, Missouri, USA

**Keywords:** influenza A virus, internalization, SLC35B4, AGRN, heparan sulfate modification, AP2B1

## Abstract

SLC35B4, a nucleotide sugar transporter that mediates the transport of UDP-GlcNAc and UDP-xylose, was found to be required for the replication of influenza A virus (IAV) of the H5N1 subtype in our genome-wide siRNA library screen. We found that defective IAV replication in SLC35B4-deficient A549 cells was independent of virus strain specificity, and the virulence of IAV in Slc35b4 knockdown mice was also decreased. By examining the individual stages of the IAV replication cycle, we discovered that the amount of internalized IAV was significantly reduced in SLC35B4-knockout A549 cells. Mechanistically, SLC35B4 facilitated IAV replication by transporting UDP-xylose, which attaches to the serine residue of heparan sulfate proteoglycans (HSPGs) in the heparan sulfate (HS) biosynthesis pathway. Knockdown of associated host factors (i.e., XYLT2, B4GALT7, EXT1, and EXT2) in the HS biosynthesis pathway also impaired IAV replication. Furthermore, we revealed that AGRN, a unique HSPG family member, was important for the endocytosis of IAV in A549 cells. Moreover, we found that the homeostasis of the AGRN protein was regulated by HS modification mediated by the initial UDP-xylose transporter SLC35B4, thereby affecting the expression level of endocytic adapter AP2B1 to influence IAV internalization. Collectively, these findings establish that SLC35B4 is an important regulator of IAV replication and uncover the underlying mechanisms by which SLC35B4 employs UDP-xylose transport activity to promote IAV internalization.

**IMPORTANCE:**

The entry process of IAV represents a favorable target for drug development. In this study, we identified SLC35B4 as an important host factor for the efficient replication of different subtypes of IAV *in vitro* and for the virulence of IAV in mice. We revealed that SLC35B4 employed its UDP-xylose transport activity to promote the HS biosynthesis pathway, thereby assisting IAV internalization into target cells in the early stage of viral infection. Consistently, several downstream factors in the HS biosynthesis pathway, i.e., XYLT2, B4GALT7, EXT1, and EXT2, as well as a specific HSPG member AGRN were also important for the replication of IAV. Furthermore, the UDP-xylose-transporting activity of SLC35B4 was involved in the regulation of the homeostasis of the AGRN protein by HS modification, which influenced virus internalization by affecting the expression levels of AP2B1. Together, the identification of the SLC35B4–XYLT2–B4GALT7–EXT1–EXT2–AGRN–AP2B1 axis may shed light on the development of potential anti-IAV therapeutics.

## INTRODUCTION

Influenza A virus (IAV) is an enveloped, single-stranded negative-sense RNA virus belonging to the Orthomyxoviridae family. It is a major threat to human health, causing seasonal epidemics and occasional global pandemics (1, 2), and also challenges the health of a variety of animals (3–5). Only 18 proteins encoded by the eight RNA segments of IAV have been identified (6). Due to the limited encoding capacity of the viral genome, IAV relies heavily on the host cellular machinery to accomplish its life cycle (7–10). As the initial step of the IAV replication cycle, the binding of viral hemagglutinin (HA) with sialic acid (SA) receptors on the cell surface triggers the receptor-mediated endocytosis of IAV (11). The endocytosis of IAV also requires supportive cellular proteins, such as clathrin, dynamin (12), Epsin-1 (13), EGFR (14), FFAR2 (15), IGDCC4 (16), PLC-γ1 (17), and mGluR2 (18). In addition to these host cellular proteins, N-glycans from glycoproteins have been implicated in the endocytosis of IAV (19). However, the roles of O-glycans (e.g., O-GlcNAc and O-xylose) in IAV endocytosis remain poorly understood.

Our previous genome-wide siRNA library screening identified the human solute carrier gene *SLC35B4* as a potential positive regulator of the replication of H5N1 virus (20). SLC35B4, belonging to the SLC35 subfamily B, maps to chromosome 7q33 in humans. It encodes a bifunctional nucleotide sugar transporter specific for the transport of UDP-N-acetylglucosamine (UDP-GlcNAc) and UDP-xylose (21). Of note, it is the only known UDP-xylose transporter. UDP-GlcNAc serves as a nucleotide sugar donor for O-GlcNAc modification (alias O-GlcNAcylation) and can be attached on the hydroxyl side chain of Ser/Thr residues of target proteins by cytoplasm-/nucleus-resident OGT (22) or ER-resident EOGT (23). UDP-xylose is attached to the Ser residues in Gly-/Ser-rich regions of proteins by xylosyltransferase I (XYLT1) and xylosyltransferase II (XYLT2) (24). The further addition of a heparan sulfate (HS) chain to the core protein [termed heparan sulfate proteoglycans (HSPGs)] is catalyzed by specific glycosyltransferases, such as B4GALT7, EXT1, and EXT2 (25, 26). UDP-GlcNAc and UDP-xylose thus act as initial substrates for O-GlcNAcylation and the HS chains of HSPGs, respectively.

O-GlcNAcylation of glycoproteins has been reported to not only promote the replication of HSV (27) but also to inhibit the replication of KSHV (28) and HIV-1 (29). The HS of HSPGs acts as a cellular receptor or co-receptor for many viruses, such as dengue virus (30), human papillomavirus (31), hepatitis C virus (32), SARS-CoV-2 (33), and human cytomegalovirus (34). However, the role of O-GlcNAc and HS modification mediated by SLC35B4 and its associated factors during IAV infection remains unknown.

In the present study, we found that the host factor SLC35B4 is required for the efficient replication of different subtypes of IAV *in vitro* and for the virulence of IAV in mice. By dissecting the viral replication cycle, we discovered that SLC35B4 mediates efficient endocytosis of IAV, which is dependent on its ability to transport UDP-xylose, but not UDP-GlcNAc. Within the family of HSPGs, AGRN acts as a unique effector to promote IAV endocytosis by interacting with the envelope protein HA and endocytic adapter AP2B1. These findings further our understanding of IAV endocytosis and support the development of antiviral countermeasures.

## RESULTS

### SLC35B4 is important for the replication of different subtypes of IAV

**Fig 1:**
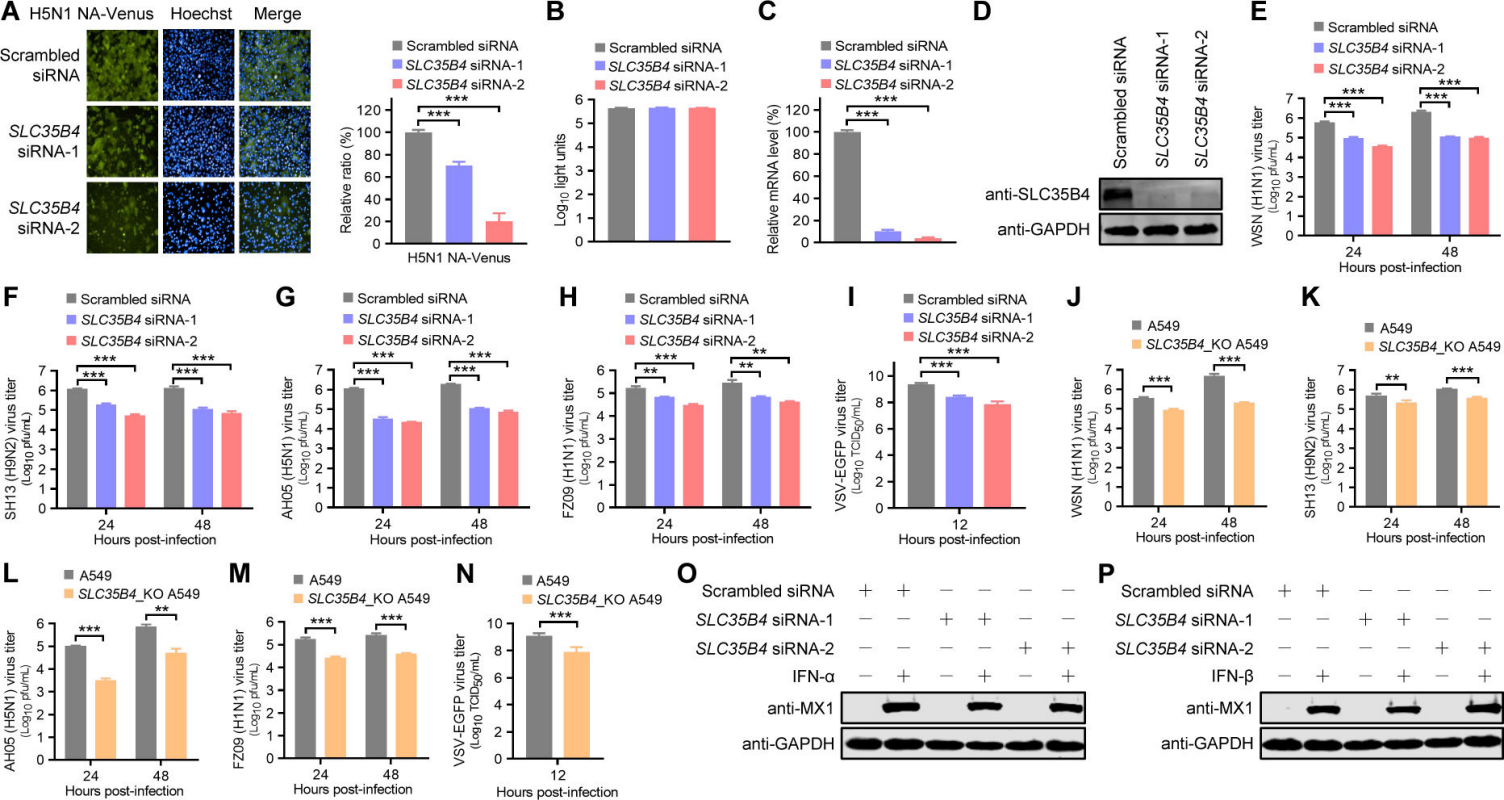
SLC35B4 positively regulates IAV and VSV replication in A549 cells. (A) Venus expression in *SLC35B4*-knockdown A549 cells infected with H5N1 NA-Venus reporter viruses (MOI = 0.1) was visualized by using the Operetta high-content imaging system at 24 h p.i. The inhibitory effect of SLC35B4 knockdown on reporter virus replication was calculated by normalizing the average fluorescence intensity of the *SLC35B4* siRNA-treated cells with that of the scrambled siRNA-treated cells (*n* = 3 biologically independent samples). ***, *P* < 0.001. (B) Viability of A549 cells transfected with *SLC35B4* siRNA or scrambled siRNA for 48 hours (*n* = 3 biologically independent samples). (C) *SLC35B4* mRNA level in *SLC35B4* siRNA-treated A549 cells determined by using RT-qPCR (*n* = 3 biologically independent samples). ***, *P* < 0.001. (D) SLC35B4 protein level in *SLC35B4* siRNA-treated A549 cells detected by Western blotting with a rabbit anti-SLC35B4 pAb. (E to I) Virus titers in *SLC35B4* siRNA- or scrambled siRNA-transfected A549 cells infected with WSN (H1N1) (MOI = 0.01) (E), SH13 (H9N2) (MOI = 0.1) (F), AH05 (H5N1) (MOI = 0.1) (G), FZ09 (H1N1) (MOI = 0.1) (H), or VSV-EGFP (100 TCID_50_) (I) virus were determined by use of plaque assays (E to H) or TCID_50_ (I) on MDCK cells at the indicated time points (*n* = 3 biologically independent samples). **, *P* < 0.01; ***, *P* < 0.001. (J to N) Virus titers in *SLC35B4*_KO or control A549 cells infected with WSN (H1N1) (MOI = 0.01) (J), SH13 (H9N2) (MOI = 0.1) (K), AH05 (H5N1) (MOI = 0.1) (L), FZ09 (H1N1) (MOI = 0.1) (M), or VSV-EGFP (100 TCID_50_) (N) virus were determined by plaque assays (J to M) or TCID_50_ (N) at the indicated time points (*n* = 3 biologically independent samples). **, *P* < 0.01; ***, *P* < 0.001. (O and P) *SLC35B4* siRNA- or scrambled siRNA-transfected A549 cells were left untreated or were treated with IFN-α (O) or IFN-β (P) for 24 hours. The cell lysates were then subjected to Western blotting with a rabbit anti-MX1 pAb for the detection of the MX1 protein.

We identified SLC35B4 as a potential proviral host factor for IAV replication by performing a whole-genome siRNA library screening targeting 21,585 human mRNAs (20), with a replication-competent Venus-expressing H5N1 virus (H5N1 NA-Venus) (35). Based on the screening results, we found that *SLC35B4* mRNA downregulation by two different siRNAs significantly reduced the fluorescence intensity produced from the growth of H5N1 NA-Venus virus in A549 cells without affecting the cell viability (Fig. 1A and B). Statistically, *SLC35B4* siRNA-2 treatment led to a higher inhibition ratio (79.6%) compared to that of *SLC35B4* siRNA-1 (29.6%) (Fig. 1A). Quantitative reverse transcription PCR (RT-qPCR) showed that the mRNA level of *SLC35B4* was reduced by approximately 90% and 95.7%, respectively, in A549 cells treated with the two specific *SLC35B4* siRNAs compared with the scrambled siRNA-treated cells (Fig. 1C). *SLC35B4*-specific siRNA-1 and -2 efficiently reduced the level of endogenous SLC35B4 protein, as determined by Western blotting (Fig. 1D).

To explore whether SLC35B4 is important for the replication of wild-type IAV strains, we infected A549 cells that were treated with *SLC35B4* siRNA with A/WSN/33 (WSN, H1N1) (MOI = 0.01), A/chicken/Shanghai/SC197/2013 (SH13, H9N2) (MOI = 0.1), A/Anhui/2/2005 (AH05, H5N1) (MOI = 0.1), or A/Fuzhou/1/2009 (FZ09, pandemic H1N1) (MOI = 0.1) virus and titrated the amount of infectious viruses in the culture supernatants by performing plaque assays on MDCK cells. We found that siRNA-mediated knockdown of SLC35B4 expression led to 6.2-/15.9- and 19.4-/21.3-fold, 6.3-/22.2- and 12.4-/19.1-fold, 35.0-/53.0- and 17.1-/26.4-fold, and 2.5-/5.5- and 4.3-/6.9-fold reductions in virus titers for WSN (H1N1), SH13 (H9N2), AH05 (H5N1), and FZ09 (H1N1) virus at 24 hours and 48 hours post-infection (p.i.), respectively (Fig. 1E through H). These results demonstrate that SLC35B4 is important for the efficient replication of different subtypes of IAVs. To assess whether SLC35B4 is specifically important for the replication of IAV, we analyzed the impact of SLC35B4 knockdown on VSV-EGFP replication. SLC35B4 knockdown also produced a 0.9–1.5 log reduction in the growth of VSV-EGFP virus at 12 h p.i. (Fig. 1I). We next generated SLC35B4 knockout (*SLC35B4*_KO) A549 cells containing a 197-bp deletion in the *SLC35B4* alleles (Fig. S1A and B), without affecting the cell viability (Fig. S1C) or morphology (Fig. S1D). Consistent with the data in SLC35B4 knockdown cells, *SLC35B4*_KO in A549 cells reduced the growth of different IAV strains at 24 h p.i. and 48 h p.i. (Fig. 1J through M) as well as the growth of VSV-EGFP virus at 12 h p.i. (Fig. 1N). Collectively, these results indicate that SLC35B4 is a host factor with broad-spectrum proviral activity.

No difference in the MX1 expression level was observed between *SLC35B4* siRNA- or scrambled siRNA-treated A549 cells upon IFN-α or IFN-β treatment (Fig. 1O and P), which indicates that SLC35B4 positively regulates IAV replication without affecting host innate immunity.

### SLC35B4 is important for the virulence of IAV in mice

**Fig 2:**
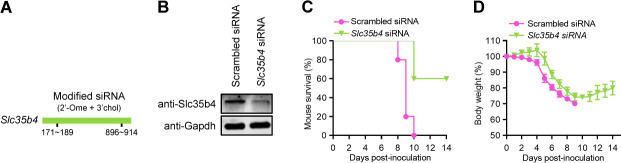
Slc35b4 is important for IAV pathogenicity in mice. (A) Schematic illustration of two 2′-O-methoxylated (2′-Ome) and 3’cholesterol (3’chol)-conjugated *Slc35b4* siRNAs used to generate *Slc35b4*_KD mice. (B) Decreased Slc35b4 expression in the lungs of *Slc35b4*_KD mice verified by Western blotting with a rabbit anti-SLC35B4 pAb. (C, D) Survival (C) and body weight changes (D) of mice (*n* = 5/group) treated with the modified scrambled siRNA and *Slc35b4* siRNA after intranasal infection with WSN (H1N1) virus (5 MLD_50_/mouse).

Given that homozygous *Slc35b4*_KO mice are nonviable due to embryonic lethality (https://www.mousephenotype.org/), we attempted to knock down the expression of Slc35b4 protein in mice (*Slc35b4*_KD) by using 2′-O-methoxylated and 3’cholesterol-conjugated *Slc35b4* siRNA to investigate the role of SLC35B4 in the virulence of IAV *in vivo* (Fig. 2A). Western blotting indicated that the level of the Slc35b4 protein in the lung extracts of *Slc35b4*_KD mice was decreased compared with that of control mice treated with modified scrambled siRNA (Fig. 2B). The mice pretreated twice with the modified *Slc35b4* siRNA or scrambled siRNA at a 36-hour interval were intranasally inoculated with five 50% mouse lethal doses (MLD_50_) of WSN (H1N1) virus, and their body weight loss and mortality were monitored daily for 14 days. We found that all five scrambled siRNA-treated mice died on days 8, 9 or 10 p.i., whereas three out of five *Slc35b4* siRNA-treated mice survived the infection (Fig. 2C). Furthermore, the average body weight of *Slc35b4* siRNA-treated mice was consistently higher than that of scrambled siRNA-treated mice during the 14-day observation period, and the surviving *Slc35b4* siRNA-treated mice began to regain body weight on day 10 p.i. (Fig. 2D). These results indicate that the decreased expression of Slc35b4 in *Slc35b4*_KD mice restricted IAV pathogenicity.

### SLC35B4 is involved in the early stage of the IAV replication cycle but has no role in the transcription or replication of the viral genome

To elucidate the mechanism by which SLC35B4 contributes to IAV replication, we first identified the specific stage of the IAV life cycle in which SLC35B4 is engaged. To this end, we validated the inhibitory effect of *SLC35B4*_KO on the levels of viral RNAs and proteins. At 4 h p.i. and 6 h p.i., the levels of NP-specific vRNA, cRNA, and mRNA were decreased by 73.3%, 82.3%, 82.1%, and 74.6%, 76.0%, 51.4%, respectively, in the *SLC35B4*_KO A549 cells compared with those in the control A549 cells (Fig. S2A and B). The expression of different viral proteins in *SLC35B4*_KO A549 cells was also impaired compared with those in control A549 cells (Fig. S2C). Therefore, the reductions in viral yield in SLC35B4-deficient A549 cells correlated with the reductions in viral RNA transcripts and protein expression.

**Fig 3:**
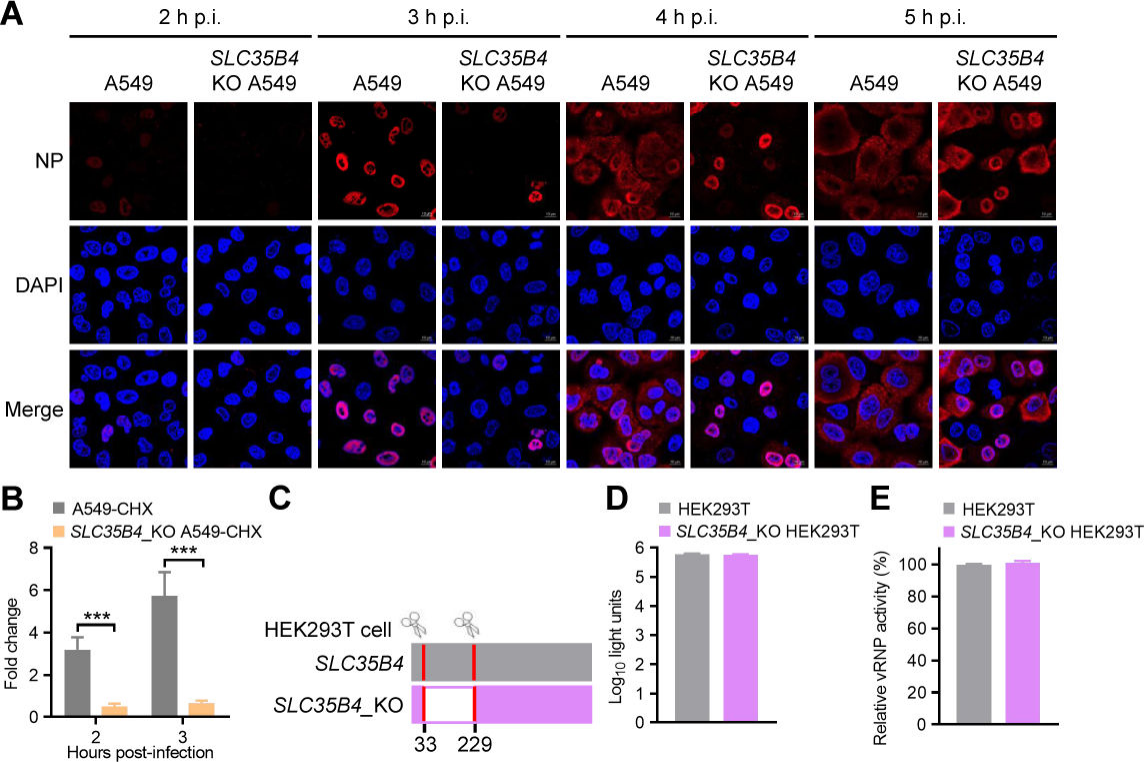
SLC35B4 affects the early stage of the IAV replication cycle. (A) *SLC35B4*_KO or control A549 cells were infected with WSN (H1N1) (MOI = 5). At 2, 3, 4, and 5 h p.i., the infected cells were fixed and stained with a mouse anti-NP mAb, followed by incubation with Alexa Fluor 633 goat anti-mouse IgG (H+L) (red). (B) *SLC35B4*_KO or control A549 cells were infected with WSN (H1N1) (MOI = 5) in the presence of cycloheximide (CHX). The levels of NP-specific vRNA in the infected cells were analyzed by RT-qPCR at 2 h p.i. and 3 h p.i. (*n* = 3 biologically independent samples). ***, *P* < 0.001. (C) Schematic diagram of two sgRNAs targeting sites at the *SLC35B4* gene loci and the corresponding truncated mutant validated by sequencing. (D) Viability of *SLC35B4*_KO HEK293T cells (*n* = 3 biologically independent samples). (E) *SLC35B4*_KO or control HEK293T cells were transfected with the four vRNP protein expression plasmids (PB2, PB1, PA, and NP) of the WSN (H1N1) virus, together with pHH21-SC09NS F-Luc and pRL-TK. At 36 hours post-transfection, a dual-luciferase reporter assay was performed in which the firefly luciferase activity was normalized to the activity of the internal Renilla luciferase control (*n* = 3 biologically independent samples).

We then investigated the NP distribution in the nucleus and cytoplasm of *SLC35B4*_KO A549 cells by using confocal imaging (Fig. 3A). At 2 h p.i. with WSN (H1N1) virus, the signal of the viral NP protein was visible in the nucleus of 27.4% of the control A549 cells, whereas it was barely detectable in *SLC35B4*_KO A549 cells. At 3 h p.i., the NP protein had accumulated in the nucleus of 72.6% of the control A549 cells, which was in clear contrast with the 17.2% nuclear accumulation in *SLC35B4*_KO A549 cells. The progression of the virus replication cycle was also restricted in *SLC35B4*_KO A549 cells compared with control cells at 4 h p.i. and 5 h p.i. In a separate experiment, we examined the amount of NP vRNA derived from the incoming vRNP complex by treating cells with cycloheximide (CHX) to prevent *de novo* synthesis of NP and found that the amount of NP vRNA in the *SLC35B4*_KO A549 cells was remarkably reduced compared with that of control A549 cells at 2 h p.i. and 3 h p.i. (Fig. 3B). Collectively, these results indicate that SLC35B4 knockout impedes the early stage of the IAV replication cycle.

*SLC35B4*_KO HEK293T cells were generated without affecting the cell viability (Fig. 3C and D). By performing a dual-luciferase reporter assay in both *SLC35B4*_KO and control HEK293T cells, we found that the vRNP activity remained unchanged in *SLC35B4*_KO HEK293T cells compared with that in control cells (Fig. 3E). Co-IP experiments showed that SLC35B4 had no direct interaction with the four vRNP proteins (Fig. S3A through D), indicating that SLC35B4 has no role in the transcription or replication of the viral genome.

Taken together, these results demonstrate that SLC35B4 is associated with an early step of the virus replication cycle, but not transcription or replication.

### SLC35B4 is not required for attachment but is important for internalization

Given that SLC35B4 is important for the early stage of the IAV replication cycle, we investigated whether it interacts with the viral envelope proteins (HA, NA, and M2) and matrix protein (M1). No interaction was observed between HA, NA, M1, or M2 and SLC35B4 (Fig. S3E through H) in Co-IP experiments, indicating that SLC35B4 does not directly interact with these viral proteins.

**Fig 4:**
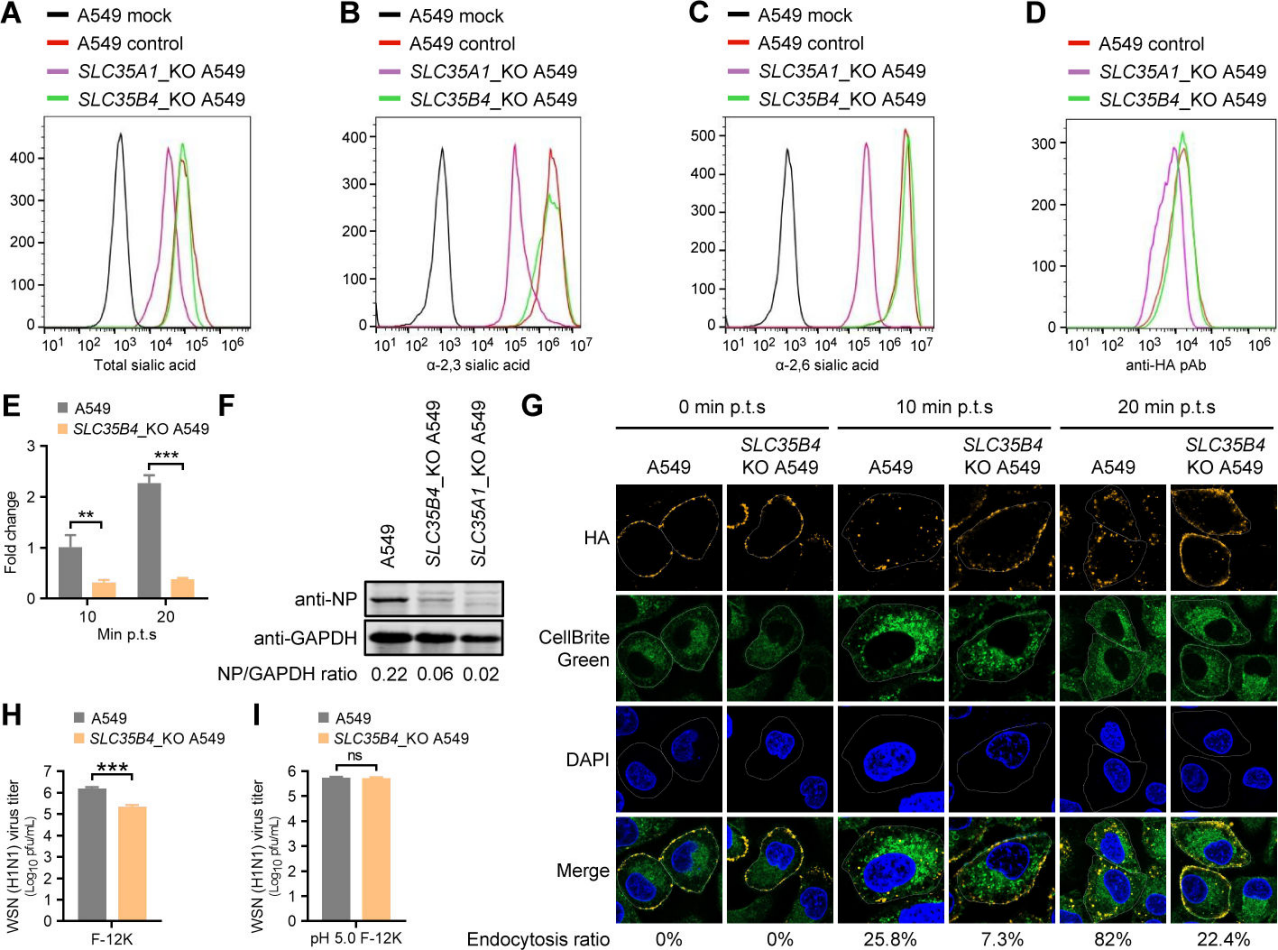
SLC35B4 is important for IAV internalization. (A to C) *SLC35B4*_KO, *SLC35A1*_KO, or control A549 cells were stained with WGA (A), MAL lectins (for α-2,3-SA) (B), or SNA lectins (for α-2,6-SA) (C) and then analyzed by flow cytometry. (D) *SLC35B4*_KO, *SLC35A1*_KO, or control A549 cells were infected with WSN (H1N1) virus (MOI = 5) at 4℃ for 1 hour. The cells were then fixed, stained with a rabbit anti-HA pAb and Alexa Fluor 488 goat anti-rabbit IgG (H+L), and analyzed by flow cytometry. (E) *SLC35B4*_KO or control A549 cells were infected with WSN (H1N1) virus (MOI = 5) at 4℃ for 1 hour, and then the temperature was shifted to 37°C. At 10 minutes or 20 minutes post temperature shift (p.t.s.), the cells were lysed after being washed with ice-cold PBS-HCl (pH 1.3), and the level of NP-specific vRNA was analyzed by RT-qPCR (*n* = 3 biologically independent samples). **, *P* < 0.01; ***, *P* < 0.001. (F) *SLC35B4*_KO, *SLC35A1*_KO, or control A549 cells were infected with WSN (H1N1) virus (MOI = 5) on ice at 4°C for 1 hour, and then the temperature was shifted to 37°C for 30 minutes. After being washed with ice-cold PBS-HCl (pH 1.3), the cells were lysed, and the amount of internalized virus particles was detected by Western blotting with a rabbit anti-NP pAb. (G) *SLC35B4*_KO or control A549 cells were infected with WSN (H1N1) virus (MOI = 10) on ice at 4°C for 1 hour and cultured at 37°C for 0 minutes, 10 minutes, or 20 minutes. The cells were fixed and stained with a rabbit anti-HA pAb (orange) and a HRP-labeled goat anti-rabbit IgG antibody according to the tyramine signal amplification protocol. The membrane was stained with CellBrite cytoplasmic membrane dyes (green). The ratio of internalized virus particles showing cytoplasmic localization was calculated from total viral particles (> 600) and is indicated at the bottom of each panel of images. (H, I) *SLC35B4*_KO or control A549 cells were infected with WSN (H1N1) virus (MOI = 1) on ice at 4°C for 1 hour and then cultured in F-12K medium (H) or acidic F-12K medium (pH 5.0) (I). After three washes with ice-cold PBS, the cells were incubated with the F-12K medium for 12 hours at 37°C. Virus titers in the supernatant were determined by use of plaque assays. ***, *P* < 0.001; ns, not significant.

IAV infection of a host cell is initiated by the binding of viral HA to SA moieties, which are terminal sugars on glycans. We, therefore, investigated whether SLC35B4 knockout changes SA expression on the cell surface. We found that the amount of SA on *SLC35B4*_KO A549 cells was indistinguishable from that on control A549 cells by using wheat germ agglutinin lectin (for total SA), *Maackia amurensis* lectin (MAL) (specific for α-2,3-SA), and *Sambucus nigra* lectin (SNA) (specific for α-2,6-SA) (Fig. 4A through C). By contrast, we observed an obvious “left shift” in the graph of SA surface expression in the positive control *SLC35A1*_KO A549 cells due to SA deficiency on the cell surface (Fig. 4A through C). These results indicate that the defective IAV replication in *SLC35B4*_KO A549 cells does not correlate with SA receptor expression on the cell surface.

Next, we assessed the effect of SLC35B4 knockout on the efficiency of viral attachment to the cell surface SA receptors. We observed a remarkable “left shift” in the graph of HA staining in the positive control *SLC35A1*_KO A549 cells, whereas there was no difference in the amount of virus adsorbed on the surface of *SLC35B4*_KO and control A549 cells (Fig. 4D), indicating that SLC35B4 knockout has no effect on IAV attachment to the cell surface.

Once the viral HA protein binds to the cell surface SA receptors, IAV rapidly initiates the endocytosis process to enter host cells. We performed an RT-qPCR analysis in *SLC35B4*_KO A549 cells and control cells that were infected with WSN (H1N1) virus on ice at 4°C for 1 hour, followed by a temperature shift to 37°C and a subsequent washing with ice-cold pH 1.3 PBS-HCl to remove the uninternalized virus (acidic PBS washing experiment). The amount of NP vRNA derived from the incoming vRNP complex was lower in *SLC35B4*_KO A549 cells compared with control cells at 10 minutes or 20 minutes post temperature shift (p.t.s.) (Fig. 4E). Next, the amount of NP in the internalized viruses was determined by Western blotting at 30 min p.t.s. in the acidic PBS washing experiment, which demonstrated that SLC35B4 knockout in A549 cells impaired virus internalization into host cells (Fig. 4F). We also performed a confocal microscopy analysis on the *SLC35B4*_KO A549 cells that were infected with the WSN (H1N1) virus (MOI = 10) on ice at 4℃ and then shifted the temperature to 37℃ for 10 minutes and 20 minutes, which directly showed that SLC35B4 knockout inhibited virus internalization rather than adsorption. The percentages of virus particles migrating from the cell membrane to the cytoplasm in normal A549 cells were 0%, 25.8%, and 82%, respectively, at 0 minutes, 10 minutes, and 20 minutes after the temperature was shifted from 4°C to 37°C (Fig. 4G). Only 7.3% and 22.4% of virus particles were internalized into the cytoplasm of *SLC35B4*_KO A549 cells at 10 minutes and 20 minutes after the temperature shift (Fig. 4G). Furthermore, we performed an acid bypass experiment (inducing direct fusion of the viral particle at the plasma membrane) to examine whether the endocytosis defect of WSN (H1N1) virus in SLC35B4_KO A549 cells could be overcome. Notably, the defect of IAV endocytosis in *SLC35B4*_KO A549 cells was observed under incubation with the normal medium but was rescued by a brief incubation in the acidic medium (pH 5.0) (Fig. 4H and I). Finally, to clarify the specificity of SLC35B4 in regulating IAV internalization, we performed a phagocytosis experiment to determine whether SLC35B4 knockout influences the total phagocytosis on the cell surface. *SLC35B4*_KO and control A549 cells were incubated with fluorescent beads at 37°C for 30 minutes, followed by flow cytometry analysis. No difference in the amount of phagocytic fluorescent beads was observed between *SLC35B4*_KO and control A549 cells (Fig. S4), indicating that SLC35B4 specifically regulates the internalization of IAV. Together, these results demonstrate that SLC35B4 is required for efficient IAV internalization into host cells.

### SLC35B4 knockout-induced endocytic retardation delays viral fusion/uncoating

Given that SLC35B4 deficiency impaired the internalization of IAV, we speculate that the progression of successive steps in the virus replication cycle may also be delayed. To test this hypothesis, we determined whether SLC35B4 knockout causes any change in the phenotype of viral fusion/uncoating. The *SLC35B4*_KO and control A549 cells were incubated with WSN (H1N1) virus (MOI = 10) on ice at 4°C for 1 hour, allowing virus binding to the cell surface, but not internalization. The culture temperature was shifted to 37°C for 1 hour to initiate viral entry in the presence of bafilomycin A1 (BafA1), an inhibitor of IAV fusion (via inhibition of vacuolar-type H+ ATPase) (36). In control A549 cells, the viral M1 signal was detected at 1 hour but not at 0 hours at 37°C, indicating that during fusion/uncoating, the M1 protein in the virus particle was free and readily detected by the M1 monoclonal antibody. As expected, the fusion/uncoating phenotype was almost imperceptible in the BafA1-treated A549 cells after the temperature shift (Fig. S5A). Notably, compared to the control A549 cells, *SLC35B4*_KO A549 cells exhibited a significant reduction in viral fusion/uncoating (Fig. S5A).

To achieve successful fusion and uncoating, the low-pH environment in late endosomes triggers conformational changes in the IAV HA protein that mediates viral fusion with the endosomal membrane (37). We labeled acidic organelles with Lysotracker (38) and found that the cells treated with the VATPase inhibitor BafA1 showed no Lysotracker signal compared with dimethyl sulfoxide (DMSO)-treated control (Fig. S5B and C). No differences in the distribution and staining intensity of the acidic compartments were observed between the *SLC35B4*_KO and control A549 cells, indicating that SLC35B4 has no role in the acidification of the endosomal compartments (Fig. S5B and C).

### SLC35B4 does not use its UDP-GlcNAc transporter function to mediate IAV endocytosis

**Fig 5:**
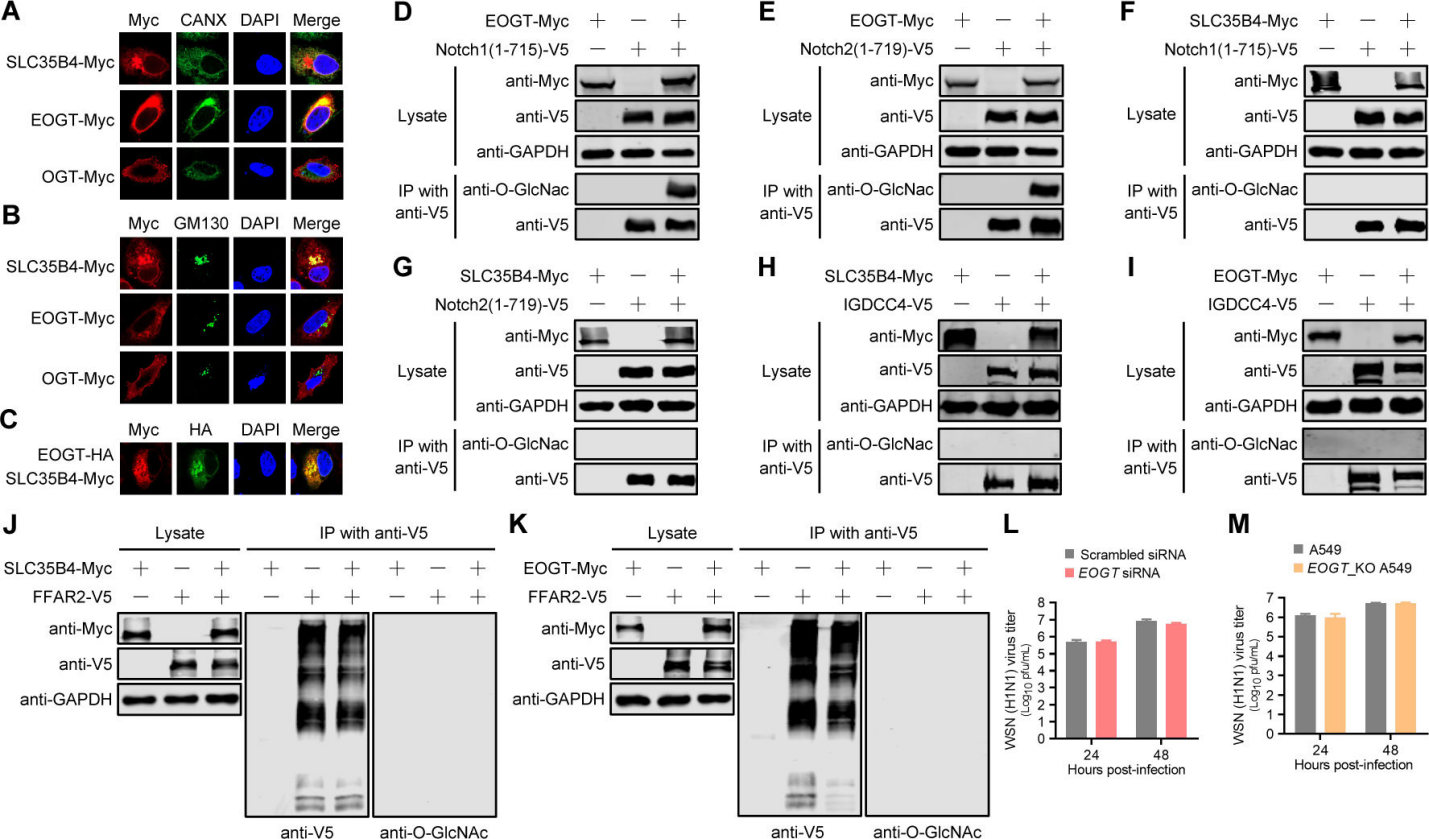
SLC35B4 mediates IAV endocytosis without using its UDP-GlcNAc transporter function. (A and B) A549 cells transfected with plasmids expressing SLC35B4-Myc, EOGT-Myc, or OGT-Myc were fixed at 24 hours post-transfection; incubated with a mouse anti-Myc mAb and a rabbit anti-CANX pAb or a rabbit anti-GM130 pAb; and stained with Alexa Fluor 633 goat anti-mouse IgG (H+L) (red) and Alexa Fluor 488 goat anti-rabbit IgG (H+L) (green). The nuclei were stained with DAPI (blue). (C) A549 cells co-transfected with plasmids expressing EOGT-HA and SLC35B4-Myc were fixed at 24 hours post-transfection, incubated with a mouse anti-HA mAb and a rabbit anti-Myc pAb, and stained with Alexa Fluor 488 goat anti-mouse IgG (H+L) (green) and Alexa Fluor 633 goat anti-rabbit IgG (H+L) (red). The nuclei were stained with DAPI (blue). (D to G) Plasmids expressing EOGT-Myc (D and E) or SLC35B4-Myc (F and G) were transfected individually or co-transfected with plasmids expressing Notch1(1-715)-V5 (D and F) or Notch2(1-719)-V5 (E and G) into HEK293T cells. At 36 hours post-transfection, the cell lysates were immunoprecipitated with a rabbit anti-V5 pAb, followed by Western blotting with a mouse anti-V5 mAb and a mouse anti-O-GlcNAc mAb for the detection of Notch1, Notch2, and their O-GlcNAc-modified forms, respectively. (H to K) Plasmids expressing SLC35B4-Myc (H and J) or EOGT-Myc (I and K) were transfected individually or co-transfected with plasmids expressing IGDCC4-V5 (H and I) or FFAR2-V5 (J and K) into HEK293T cells. At 36 hours post-transfection, the cell lysates were immunoprecipitated with a rabbit anti-V5 pAb, followed by Western blotting with a mouse anti-V5 mAb and a mouse anti-O-GlcNAc mAb for the detection of IGDCC4, FFAR2, and their O-GlcNAc-modified forms, respectively. (L and M) Virus titers in *EOGT*-knockdown or *EOGT*_KO A549 cells infected with the WSN (H1N1) virus (MOI = 0.01) at the indicated time points, as determined by using plaque assays on MDCK cells (*n* = 3 biologically independent samples).

The function of proteins usually correlates with their localization in cells. We found that SLC35B4 is distributed in the ER and Golgi apparatus of A549 cells (Fig. 5A and B). This finding suggests that the role of SLC35B4 in promoting the internalization of IAV is achieved indirectly.

SLC35B4 is known to transport cytoplasmic UDP-GlcNAc into the lumen of the ER and the Golgi apparatus for glycosyltransferases (21). In mammals, UDP-GlcNAc serves as a sugar donor for two kinds of O-GlcNAc transferases termed cytosolic/nuclear enzyme OGT and ER-resident enzyme EOGT. We found that EOGT only colocalized with CANX (an ER marker protein), and OGT was distributed throughout the cytoplasm (Fig. 5A and B). SLC35B4 and EOGT were obviously colocalized in the ER (Fig. 5C). EOGT is responsible for the addition of UDP-GlcNAc to secreted and membrane proteins containing one or more epidermal growth factor-like repeats with a specific consensus sequence (23). EOGT-mediated O-GlcNAc modification of Notch1 and Notch2 containing epidermal growth factor-like repeats was readily detected in HEK293T cells (Fig. 5D and E), indicating that EOGT has UDP-GlcNAc transferase activity. By contrast, SLC35B4 overexpression could not mediate O-GlcNAc modification of Notch1 and Notch2 (Fig. 5F and G). FFAR2 and IGDCC4 have previously been revealed to play important roles in IAV internalization (15, 16). Co-IP analysis showed that FFAR2 and IGDCC4 were not O-GlcNAc-modified under the conditions of SLC35B4 or EOGT overexpression (Fig. 5H through K). We also found that the growth of the WSN (H1N1) virus in EOGT-silenced A549 cells was comparable to that in A549 cells transfected with scrambled siRNA (Fig. 5L; Fig. S6A through C). In addition, there were no differences in progeny virus titers between *EOGT*_KO and control A549 cells (Fig. 5M; Fig. S6D through G). Overall, these results indicate that the UDP-GlcNAc transporter/transferase function of SLC35B4/EOGT is not associated with IAV endocytosis.

### SLC35B4-mediated transportation of UDP-xylose is important for IAV replication

**Fig 6:**
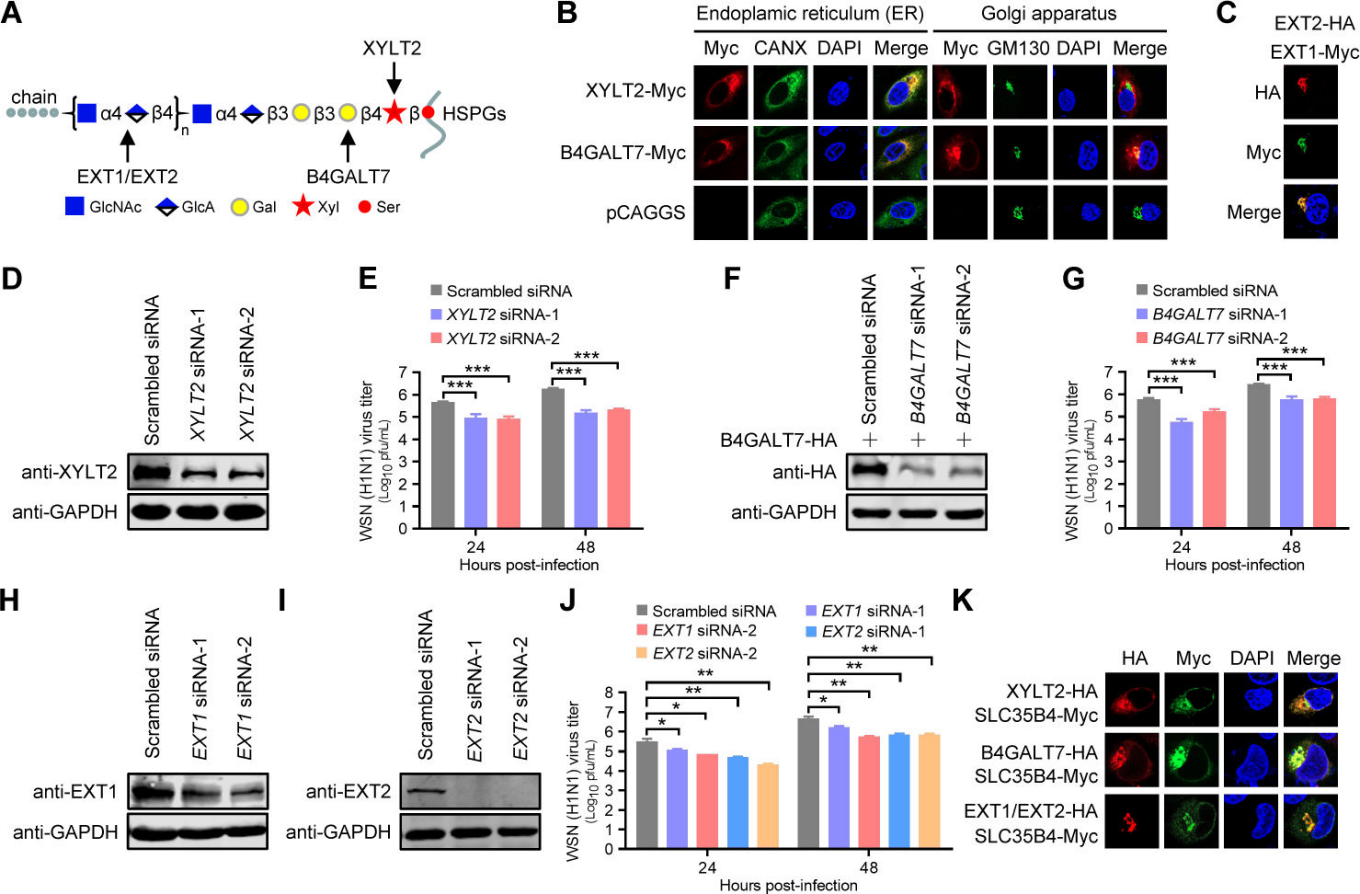
O-xylose-associated factors promote IAV replication in A549 cells. (A) Schematic diagram of HS chain elongation of heparan sulfate proteoglycans (HSPGs). Xyl, xylose; Gal, galactose; GlcA, glucuronic acid; GlcNAc, *N*-acetylglucosamine. (B and C) A549 cells transfected with plasmids expressing XYLT2-Myc or B4GALT7-Myc (B) or co-transfected with plasmids expressing EXT1-Myc and EXT2-HA (C) were fixed at 24 hours post-transfection, incubated with a mouse anti-Myc mAb and a rabbit anti-CANX pAb or a rabbit anti-GM130 pAb (B) or with a mouse anti-HA mAb and a rabbit anti-Myc pAb (C), and stained with Alexa Fluor 633 goat anti-mouse IgG (H+L) (red) and Alexa Fluor 488 goat anti-rabbit IgG (H+L) (green). (D) The XYLT2 protein level was detected by Western blotting in A549 cells transfected with *XYLT2* siRNA or scrambled siRNA for 48 hours. (E) Virus titers in *XYLT2*-knockdown A549 cells infected with the WSN (H1N1) virus (MOI = 0.01) at the indicated time points (*n* = 3 biologically independent samples). ***, *P* < 0.001. (F) A549 cells were transfected with *B4GALT7* siRNA or scrambled siRNA for 12 hours and further transfected with plasmids expressing B4GALT7-HA for 24 hours. The B4GALT7 protein level was evaluated by Western blotting. (G) Virus titers in *B4GALT7*-knockdown A549 cells infected with the WSN (H1N1) virus (MOI = 0.01) at the indicated time points (*n* = 3 biologically independent samples). ***, *P* < 0.001. (H, I) EXT1 (H) or EXT2 (I) protein levels detected by Western blotting in A549 cells transfected with *EXT1* siRNA, *EXT2* siRNA, or scrambled siRNA for 48 hours. (J) Virus titers in *EXT1*- or *EXT2*-knockdown A549 cells infected with WSN (H1N1) virus (MOI = 0.01) at the indicated time points (*n* = 3 biologically independent samples). *, *P* < 0.05; **, *P* < 0.01. (K) A549 cells co-transfected with plasmids expressing SLC35B4-Myc and XYLT2-HA, B4GALT7-HA, or EXT1/EXT2-HA were fixed at 24 hours post-transfection, incubated with a mouse anti-HA mAb and a rabbit anti-Myc pAb, and stained with Alexa Fluor 633 goat anti-mouse IgG (H+L) (red) and Alexa Fluor 488 goat anti-rabbit IgG (H+L) (green).

SLC35B4 is also involved in the transport of UDP-xylose (21). ER-resident XYLT2 (Fig. 6A and B) catalyzes the conjugation of UDP-xylose to specific Ser residues of the core protein and initiates the biosynthesis of glycosaminoglycan (GAG) chains in proteoglycans (24). The *XYLT2*-specific siRNA-1 and -2 efficiently reduced the XYLT2 expression level, as determined by RT-qPCR (Fig. S7A) and Western blotting (Fig. 6D), without affecting the viability of A549 cells (Fig. S7B). XYLT2 downregulation in A549 cells by two different siRNAs reduced the growth of WSN (H1N1) virus by 5.1-/11.7-fold (*XYLT2* siRNA-1) or 5.5-/8.2-fold (*XYLT2* siRNA-2) at 24 h p.i./48 h p.i. compared to scrambled siRNA-transfected cells (Fig. 6E).

During GAG synthesis, the second galactose (Gal) residue is added in the tetrasaccharide linkage region (GlcAβ1-3Galβ1-3Galβ1-4Xylβ1-) by the ER-/Golgi-resident B4GALT7 (Fig. 6A and B) (39). Similar to the observation in XYLT2-silenced A549 cells, B4GALT7-silencing (Fig. 6F; Fig. S7C) had no cytotoxic effects on A549 cells (Fig. S7D) and reduced the growth of the WSN (H1N1) virus (MOI = 0.01) by 10.3-/4.7- or 3.4-/4.3-fold at 24 h p.i./48 h p.i., respectively (Fig. 6G). These results further indicate that IAV is dependent upon the GAG biosynthesis pathway for efficient infection.

EXT1 and EXT2 formed a Golgi apparatus-located hetero-oligomeric complex in A549 cells (Fig. 6C), which catalyzes the chain elongation step in HS biosynthesis of proteoglycans (26). The corresponding specific siRNA treatment efficiently reduced the expression of EXT1 or EXT2 compared with scrambled siRNA without affecting the cell viability (Fig. 6H and I; Fig. S7E through G). SiRNA-mediated knockdown of EXT1 or EXT2 decreased the production of infectious viruses at 24 h p.i./48 h p.i. (Fig. 6J). Furthermore, confocal microscopy analysis showed that the four HS metabolism-associated genes (XYLT2, B4GALT7, EXT1, and EXT2) could colocalize with SLC35B4 (Fig. 6K). Collectively, these data clearly indicate that xylose utilization-associated factors are involved in the regulation of IAV replication.

### AGRN is important for IAV internalization

**Fig 7:**
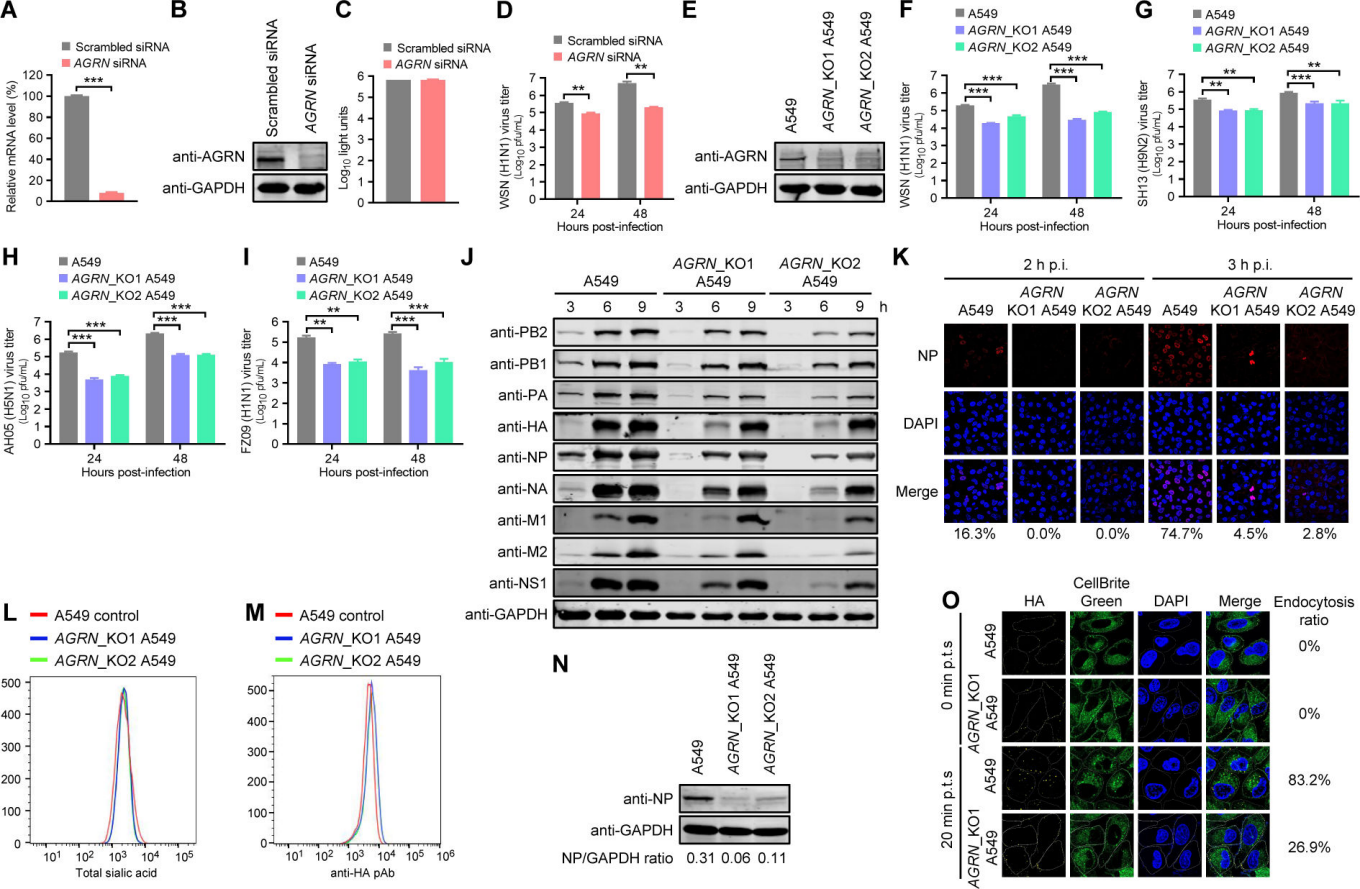
AGRN is important for IAV endocytosis. (A and B) Detection of the *AGRN* mRNA level or protein expression in *AGRN*-knockdown A549 cells by using RT-qPCR (A) (*n* = 3 biologically independent samples) or Western blotting (B). ***, *P* < 0.001. (C) Viability of A549 cells transfected with *AGRN* siRNA or scrambled siRNA for 48 hours (*n* = 3 biologically independent samples). (D) Virus titers in *AGRN*-knockdown A549 cells infected with the WSN (H1N1) virus (MOI = 0.01) at the indicated time points (*n* = 3 biologically independent samples). **, *P* < 0.01. (E) AGRN protein level detected by Western blotting in *AGRN*_KO A549 cells. (F to I) Virus titers in *AGRN*_KO or control A549 cells infected with WSN (H1N1) (MOI = 0.01) (F), SH13 (H9N2) (MOI = 0.1) (G), AH05 (H5N1) (MOI = 0.1) (H), or FZ09 (H1N1) (MOI = 0.1) (I) virus at the indicated time points (*n* = 3 biologically independent samples). **, *P* < 0.01; ***, *P* < 0.001. (J) Expression of viral proteins in *AGRN*_KO A549 cells infected with WSN (H1N1) virus (MOI = 5) was detected by Western blotting at 3 h p.i., 6 h p.i., and 9 h p.i. (K) *AGRN*_KO and control A549 cells were infected with the WSN (H1N1) virus (MOI = 5). At 2 h p.i. and 3 h p.i., the cells were fixed and stained with a mouse anti-NP mAb and Alexa Fluor 633 goat anti-mouse IgG (H+L) (red). (L) *AGRN*_KO or control A549 cells were stained with WGA and analyzed by flow cytometry. (M) *AGRN*_KO or control A549 cells were infected with WSN (H1N1) virus (MOI = 5) at 4°C for 1 hour. The cells were fixed and stained with a rabbit anti-HA pAb and Alexa Fluor 488 goat anti-rabbit IgG (H+L) and analyzed by flow cytometry. (N) *AGRN*_KO or control A549 cells were infected with the WSN (H1N1) virus (MOI = 5) on ice at 4°C for 1 hour, and then the temperature was shifted to 37°C for 30 minutes. After being washed with ice-cold PBS-HCl (pH 1.3), the cells were lysed, and the amount of internalized virus particles was evaluated by detecting the viral NP protein by Western blotting. (O) *AGRN*_KO1 or control A549 cells were infected with the WSN (H1N1) virus (MOI = 10) on ice at 4°C for 1 hour and cultured at 37°C for 0 or 20 minutes. The cells were fixed and stained with a rabbit anti-HA pAb and HRP-labeled goat anti-rabbit IgG antibody according to the tyramine signal amplification protocol.

HSPGs comprise glycoproteins that are characterized by a core protein containing at least one covalently attached HS chain (40). There are three subfamilies of HSPGs: membrane HSPGs (SDC1~SDC4, GPC1~GPC6, TGFBR3, NRP1, and CD44v3), secreted extracellular matrix HSPGs (AGRN, HSPG2, and COL8A1), and secretory vesicle proteoglycan (SRGN) (41). Based on the expression abundance and subcellular localization of these genes (https://www.proteinatlas.org/) in A549 cells, we selected SDC1, GPC1, TGFBR3, NRP1, AGRN, and HSPG2 to explore their effects on IAV replication. RT-qPCR and Western blotting revealed that treatment with specific siRNAs efficiently reduced the expression of the corresponding genes compared with scrambled siRNA (Fig. 7A and B; Fig. S7H), without affecting the cell viability (Fig. 6C; Fig. S7I). Among these HSPGs, only AGRN knockdown decreased the production of infectious WSN (H1N1) virus by 4.0-/24.0-fold at 24 h p.i./48 h p.i. (Fig. 7D; Fig. S7J). To validate the siRNA interference results, we evaluated the effect of AGRN knockout on IAV replication. Because the AGRN gene has a 6,207-nucleotide sequence, we employed a CRISPR/Cas9 strategy with the use of a pair of gRNAs targeting the N-terminal AGRN or a single gRNA targeting the C-terminal AGRN to generate two types of *AGRN*_KO A549 cells that had a 426-bp or 1-bp deletion (Fig. S8A), leading to the loss of AGRN protein expression (Fig. 7E). AGRN knockout had no effect on the cell viability or morphology (Fig. S8B and C). Importantly, AGRN knockout in *AGRN*_KO1 A549 cells and *AGRN*_KO2 A549 cells led to 10.0-/103.4- and 3.9-/35.4-fold, 4.2-/4.2- and 4.1-/4.0-fold, 35.3-/17.6- and 22.1-/16.3-fold, and 21.2-/63.1- and 15.5-/25.6-fold decreases in the growth titers of WSN (H1N1), SH13 (H9N2), AH05 (H5N1), and FZ09 (H1N1) virus at 24 h p.i./48 h p.i., respectively (Fig. 7F through I), demonstrating that AGRN knockout appears to inhibit the replication of diverse subtypes of IAV. In addition, an overall slowdown of viral protein accumulation in *AGRN*_KO A549 cells was detected compared with the normal A549 cells by Western blotting (Fig. 7J), indicating a delayed viral replication cycle. This observation was supported by confocal imaging: the nucleus-localized ratio of the NP protein in WSN (H1N1)-infected *AGRN*_KO1 and *AGRN*_KO2 A549 cells was 0%/4.5% and 0%/2.8% at 2 h p.i./3 h p.i., compared with 16.3%/74.7% in control cells at the same time points, respectively (Fig. 7K).

To identify the early step(s) at which AGRN engages, we first clarified that AGRN knockout had no effect on the expression of gross SA on the cell surface (Fig. 7L). A virus attachment assay showed no difference in the amount of viruses adsorbed on *AGRN*_KO and control A549 cells (Fig. 7M), indicating that AGRN has no effect on the attachment of IAV to the cell surface. We then investigated whether AGRN knockout inhibits the subsequent internalization step by performing an acidic PBS washing experiment. AGRN knockout remarkably inhibited IAV endocytosis (Fig. 7N). To further corroborate the role of AGRN in IAV internalization, *AGRN*_KO or control A549 cells were infected with the WSN (H1N1) virus (MOI = 10) on ice at 4°C for 1 hour, followed by a temperature shift to 37°C for 0 minutes and 20 minutes. We found that the virus particles were attached to the plasma membrane of *AGRN*_KO A549 cells and control A549 cells after incubation at 4°C for 1 hour (Fig. 7O). When the culture temperature was shifted to 37°C and kept for 20 minutes, 83.2% of the virus particles were internalized into the cytoplasm of the control A549 cells, but only 26.9% of the virus particles were internalized into the cytoplasm of the *AGRN*_KO A549 cells (Fig. 7O). Taken together, these results demonstrate that AGRN is a key cellular factor for the efficient internalization of IAV into host cells.

### The 30-350 and 1325-1548 regions of AGRN mediate its interaction with the IAV HA protein

**Fig 8:**
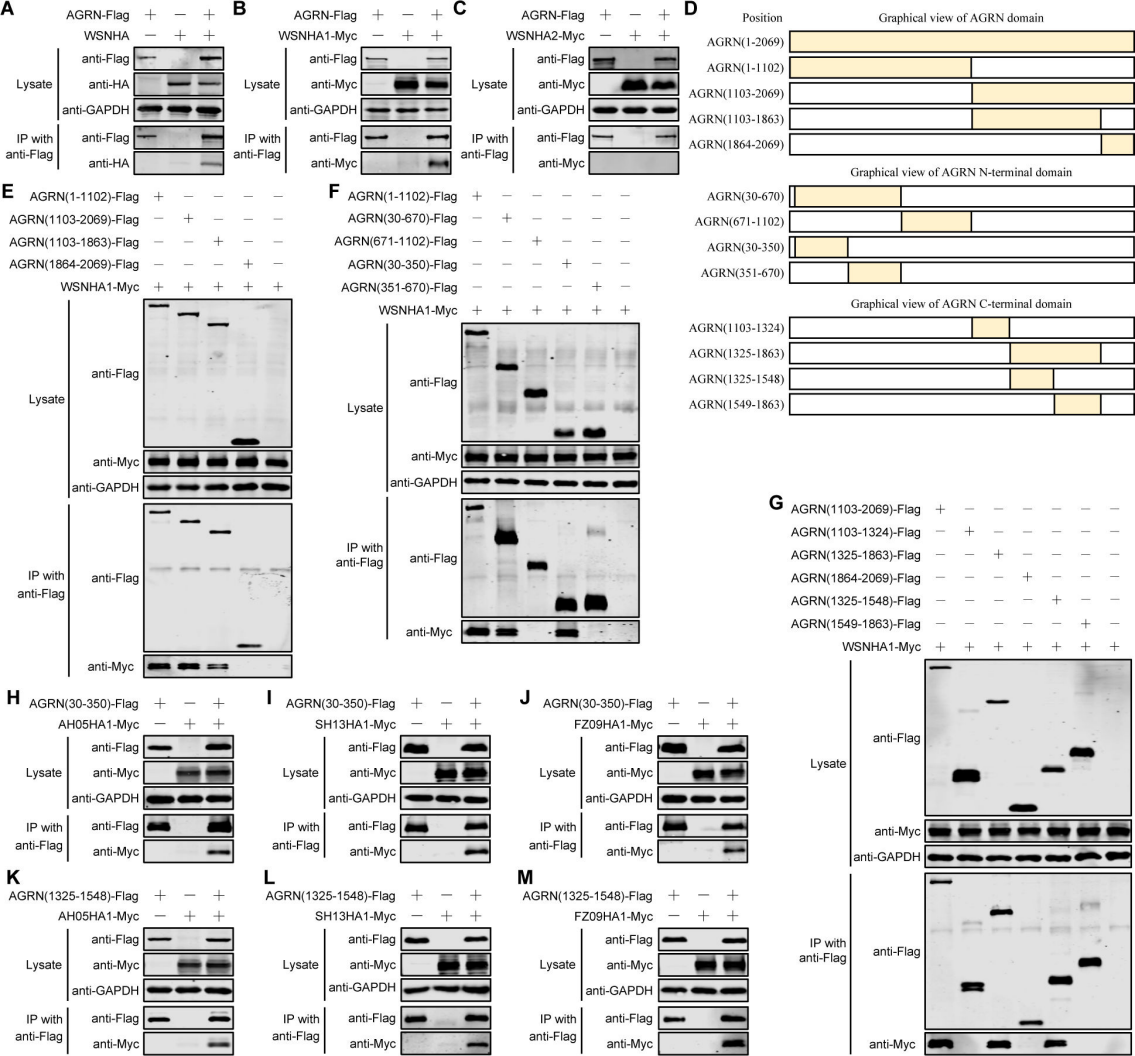
The 30–350 and 1325–1548 regions of AGRN interact with IAV HA1. (A to C) Plasmids expressing AGRN-Flag were transfected individually or co-transfected with plasmids expressing HA (A) or Myc-tagged HA1 (B) or HA2 (C) of the WSN (H1N1) virus into HEK293T cells. At 36 hours post-transfection, cell lysates were immunoprecipitated with a mouse anti-Flag mAb and subjected to Western blotting with the indicated antibodies. (D) Schematic diagram of AGRN(1–2069) truncation mutants. (E to G) Plasmids expressing AGRN-Flag truncation mutants (E), AGRN(1–1102)-Flag truncation mutants (F), or AGRN(1103–2069)-Flag truncation mutants (G) were transfected individually or co-transfected with plasmids expressing HA1-Myc of the WSN (H1N1) virus into HEK293T cells. At 36 hours post-transfection, Co-IP and Western blotting were carried out as described in (A to C). (H to M) Plasmids expressing AGRN(30-350)-Flag (H to J) or AGRN(1325–1548)-Flag (K to M) were transfected individually or co-transfected with plasmids expressing Myc-tagged HA1 of AH05 (H5N1) (H and K), SH13 (H9N2) (I and L), or FZ09 (H1N1) (J and M) viruses into HEK293T cells. At 36 hours post-transfection, Co-IP and Western blotting were carried out as described in (A to C).

Of the three envelope proteins (HA, NA, and M2) of IAV, HA plays a major role in viral invasion. To further explore the molecular mechanisms by which AGRN regulates IAV internalization, we first examined whether AGRN binds to the HA protein. We found that AGRN had an obvious interaction with WSN (H1N1) HA (Fig. 8A). To further determine which specific region of HA is essential for its interaction with AGRN, we constructed the HA1 and HA2 domains of WSN (H1N1) HA and evaluated their interaction with AGRN by Co-IP experiments. We found that the HA1 domain was responsible for the association with AGRN (Fig. 8B and C).

AGRN comprises an N-terminal 110-kDa subunit (1–1102) and a C-terminal 110-kDa subunit (1103–2069); this latter subunit is further divided into a C-terminal 90-kDa subunit (1103–1863) and a C-terminal 20-kDa subunit (1864–2069) (Fig. 8D). To map the AGRN subunit involved in binding to HA1 of the WSN (H1N1) virus, we constructed four Flag-tagged AGRN truncation mutants. Co-IP assays in HEK293T cells indicated that the N-terminal 110-kDa subunit (1–1102) and C-terminal 90-kDa subunit (1103–1863) both interacted with HA1 (Fig. 8E). We next attempted to identify the specific regions in these two AGRN subunits that are critical for their binding to HA1. To this end, we generated four truncated constructs of the N-terminal 110-kDa subunit (1–1102) [AGRN(30–670), AGRN(671–1102), AGRN(30–350), and AGRN(351–670)] and four truncated constructs of the C-terminal 90-kDa subunit (1103–1863) [AGRN(1103–1324), AGRN(1325–1863), AGRN(1325–1548), and AGRN(1549–1863)] with a Flag tag at the C-terminus (Fig. 8D) and then examined their interaction with the HA1 domain in HEK293T cells by Co-IP experiments. Ultimately, we found that the AGRN(30–350) and AGRN(1325–1548) regions showed strong binding with the HA1 domain (Fig. 8F and G).

The interactive region (30–350 aa) of AGRN with HA1 contains two of four N-linked glycosylation sites (positions 135 and 250) (www.uniprot.org), that are potentially modified by SA. To determine whether the AGRN–HA1 interaction is mediated by SA, we generated a *SLC35A1*_KO HEK293T cell line (Fig. S9A through D) that is deficient in SA synthesis (42). By performing Co-IP experiments, we found that the interaction between AGRN(30–350) and HA1 was similarly observed in *SLC35A1*_KO or control HEK293T cells (Fig. S9E), indicating that the AGRN–HA1 interaction does not require the involvement of SA.

To further determine whether the HA1-binding regions of AGRN also mediate the interaction with the HA1 of different IAV strains, we performed Co-IP experiments to examine the interactions between the AGRN(30–350) or AGRN(1325–1548) region with Myc-tagged HA1 of IAV strains other than WSN (H1N1). We found that AGRN(30–350) (Fig. 8H through J) and AGRN(1325–1548) (Fig. 8K through M) interacted with HA1 of AH05 (H5N1), SH13 (H9N2), and FZ09 (H1N1) viruses. These results indicate that AGRN appears to mediate a broad-spectrum interaction with the HA1 of different subtypes of IAV.

### SLC35B4-mediated HS modification of AGRN promotes internalization of IAV by regulating the stability of endocytic adapter AP2B1

**Fig 9:**
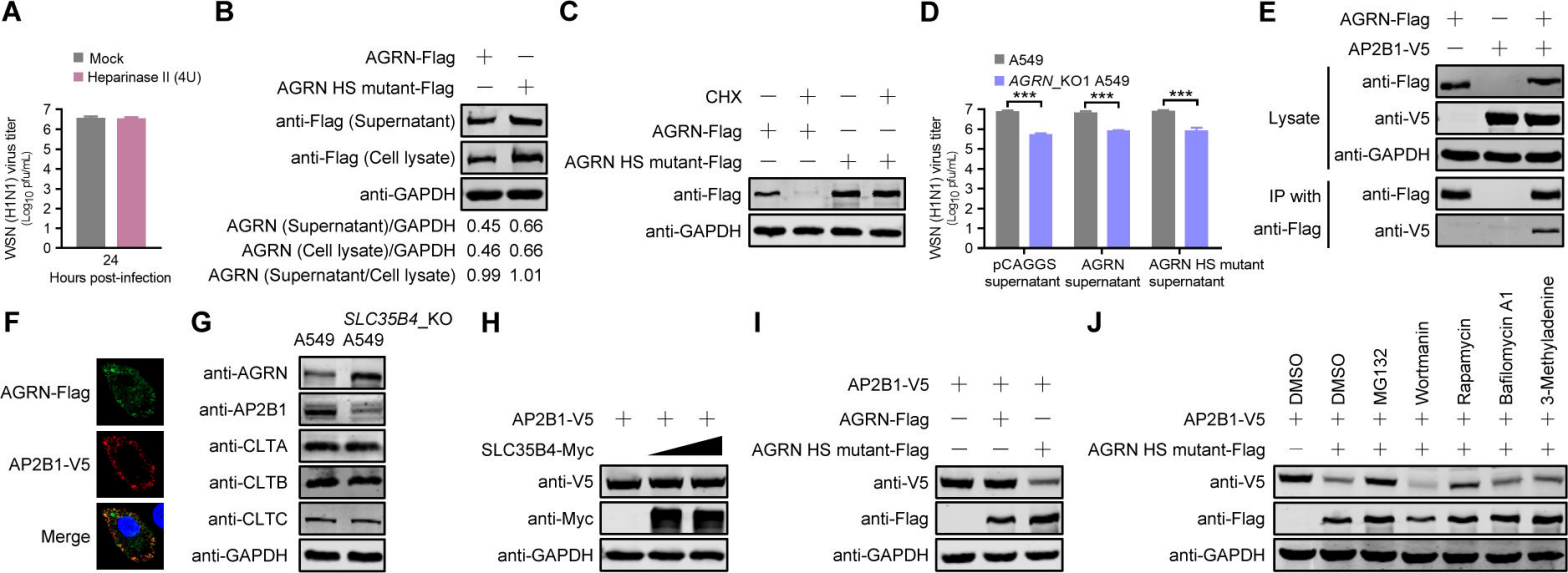
HS modification of AGRN promotes IAV internalization by regulating the stability of endocytic adapter AP2B1. (A) Virus titers in heparinase II (4U)-treated A549 cells infected with the WSN (H1N1) virus (MOI = 0.01) were determined by plaque assays at 24 h p.i. (*n* = 3 biologically independent samples). (B) Plasmids expressing AGRN-Flag or AGRN HS mutant-Flag were transfected into HEK293T cells. Cell lysates and supernatants were subjected to Western blotting with a mouse anti-Flag mAb at 36 hours post-transfection. (C) HEK293T cells were transfected with plasmids expressing AGRN-Flag or AGRN HS mutant-Flag. At 36 hours post-transfection, the cells were treated with CHX for 10 hours, and then cell lysates were subjected to Western blotting with a mouse anti-Flag mAb. (D) *AGRN*_KO1 or control A549 cells were infected with WSN (H1N1) virus (MOI = 0.01) that was pre-incubated at 4°C for 1 hour with the supernatant collected from HEK293T cells transfected with plasmids expressing AGRN-Flag, AGRN HS mutant-Flag or the pCAGGS vector. Virus titers were determined by plaque assays at 24 h p.i. (*n* = 3 biologically independent samples). ***, *P* < 0.001. (E) HEK293T cells were transfected with the indicated combinations of plasmids expressing AGRN-Flag and AP2B1-V5. At 36 hours post-transfection, cell lysates were immunoprecipitated with a mouse anti-Flag mAb and then subjected to Western blotting with the indicated antibodies. (F) A549 cells co-transfected with plasmids expressing AGRN-Flag and AP2B1-V5 were fixed at 24 hours post-transfection, incubated with a mouse anti-Flag mAb and a rabbit anti-V5 pAb, and stained with Alexa Fluor 488 goat anti-mouse IgG (H+L) (green) and Alexa Fluor 633 goat anti-rabbit IgG (H+L) (red). (G) Endogenous expressions of AGRN, AP2B1, clathrin subunits (CLTA, CLTB, and CLTC) in A549 cells and *SLC35B4*_KO A549 cells by Western blotting with the indicated antibodies. (H, I) HEK293T cells were transfected with plasmids expressing AP2B1-V5 individually or in combination with plasmids expressing SLC35B4-Myc (H), AGRN-Flag, or AGRN HS mutant-Flag (I). At 36 hours post-transfection, cell lysates were subjected to Western blotting. (J) HEK293T cells were transfected with plasmids expressing AP2B1-V5 individually or in combination with plasmids expressing AGRN HS mutant-Flag. At 36 hours post-transfection, the cells were treated with proteasome inhibitor (MG132) or autophagy inhibitors (wortmannin, rapamycin, bafilomycin A1 or 3-methyladenine) for 24 hours. Cell lysates were subjected to Western blotting.

HS acts as an early attachment factor to initiate the endocytosis of numerous viruses (30–34). To investigate whether the HS of the plasma membrane promotes the replication of IAV, A549 cells were pretreated with heparinase to remove HS from the plasma membrane and subsequently infected with WSN (H1N1) virus (MOI = 0.01). As shown in Fig. 9A, the virus growth was not susceptible to heparinase treatment, indicating that SLC35B4-mediated HS modification does not directly affect the attachment of IAV. To determine whether AGRN is a substrate for the HS modification catalyzed by XYLT2, B4GALT7, and EXT1, we performed Co-IP experiments to examine the interactions between AGRN and these catalyzing molecules of HS synthesis. We found that AGRN had obvious interactions with XYLT2, B4GALT7, or EXT1 (Fig. S10A through C), implying that AGRN is a HSPG subjected to HS modification.

Next, we examined whether SLC35B4-mediated HS modification affects the secretion and stability of the AGRN protein. Since the AGRN protein contains several Gly-/Ser-rich regions and SGXG motifs as putative GAG attachment sites, we first generated an AGRN HS mutant (harboring 15 Ser to Ala mutations) with a Flag tag at the C-terminus. HEK293T cells were transfected with plasmids expressing AGRN-Flag or AGRN HS mutant-Flag. Surprisingly, the expression of the AGRN HS mutant was apparently higher than that of wild-type AGRN in both the cell lysate and supernatant, and the level of secreted AGRN in the supernatant was well correlated with that of AGRN in the cell lysate (Fig. 9B). Meanwhile, the stability of the AGRN HS mutant was increased compared with that of wild-type AGRN in the presence of cycloheximide (CHX) to inhibit protein synthesis (Fig. 9C). These results indicate that HS modification regulates the expression and turnover of AGRN, but not its secretion across the plasma membrane. Subsequently, we investigated whether the secreted AGRN could rescue the growth defects of IAV in *AGRN*_KO A549 cells. We collected the supernatant from HEK293T cells transfected with plasmids expressing AGRN-Flag, AGRN HS mutant-Flag, or empty pCAGGS vector and then incubated the supernatant with the WSN (H1N1) virus (MOI = 0.01) at 4°C for 1 hour. After pre-incubation, the mixtures were used to infect *AGRN*_KO or control A549 cells, and virus titers were determined by performing plaque assays on MDCK cells. We found that the complement of secreted AGRN or AGRN HS mutant did not rescue the virus growth defects in *AGRN*_KO A549 cells (Fig. 9D), indicating that SLC35B4-mediated HS modification of AGRN does not regulate IAV endocytosis by influencing the level of secreted AGRN.

When IAV invades host cells, it recruits endocytic adapters clathrin (CLTA, CLTB, and CLTC) and AP2B1 to initiate virus endocytosis (15, 18). To investigate whether AGRN is involved in the recruitment of these downstream endocytic adapters, HEK293T cells were transfected with plasmids expressing AGRN-Flag and AP2B1-V5 or Myc-tagged CLTA, CLTB, or CLTC. The Co-IP assay showed that AGRN only interacted with AP2B1 (Fig. 9E; Fig. S11A through C). Meanwhile, punctate co-localization between AGRN-Flag and AP2B1-V5 was displayed in the plasma membrane of co-transfected A549 cells by confocal microscopy (Fig. 9F). To ascertain the relationship between SLC35B4-mediated HS modification of AGRN and AP2B1, we detected the endogenous levels of AGRN and AP2B1 in the *SLC35B4*_KO A549 cells. Notably, we found that the deficiency of HS modification of AGRN induced by SLC35B4 knockout led to an increase in endogenous AGRN expression but a decrease in endogenous AP2B1 expression (Fig. 9G). To explore whether SLC35B4, AGRN, or AGRN HS mutant directly mediates AP2B1 degradation, HEK293T cells were transfected with different combinations of plasmids expressing AP2B1-V5 and SLC35B4-Myc, AGRN-Flag, or AGRN HS mutant-Flag. We found that overexpression of SLC35B4 or AGRN had no effect on the levels of AP2B1, but the HS mutation of AGRN caused excessive accumulation of the AGRN HS mutant, which further led to the degradation of AP2B1 (Fig. 9H and I). To investigate the specific pathway by which the AGRN HS mutant mediated the degradation of AP2B1, HEK293T cells were co-transfected to express AP2B1 and AGRN HS mutant, followed by treatment with proteasome inhibitor (MG132) or autophagy inhibitors (wortmannin, rapamycin, bafilomycin A1 or 3-methyladenine). We found that only MG132 treatment prevented AP2B1 degradation in the presence of the overexpressed AGRN HS mutant (Fig. 9J), indicating that the AGRN HS mutant mediates the degradation of AP2B1 via the proteasome pathway.

**Fig 10:**
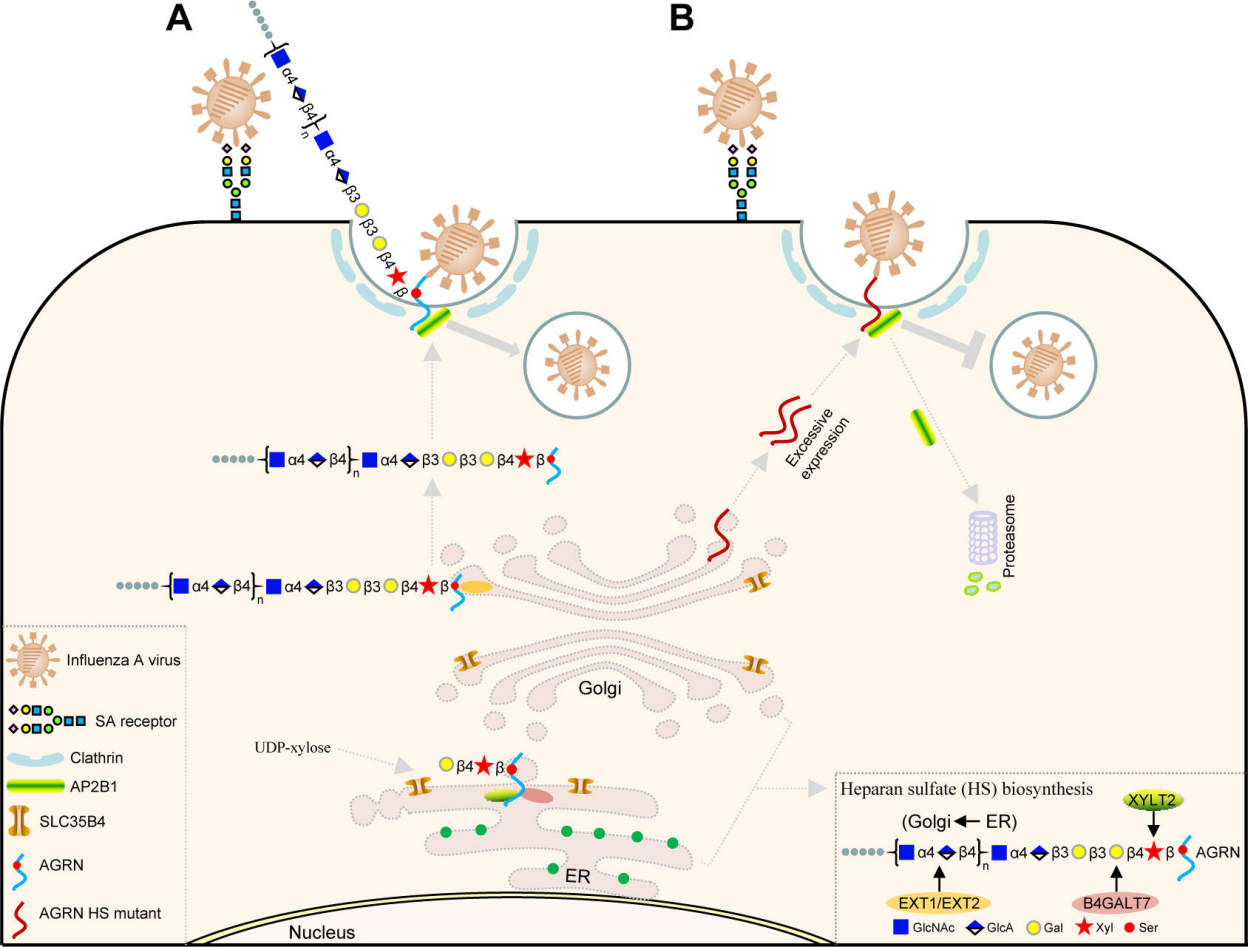
SLC35B4-mediated HS modification of AGRN promotes IAV internalization into host cells. (A) SLC35B4 transports UDP-xylose from the cytoplasm to the endoplasmic reticulum (ER), XYLT2 catalyzes UDP-xylose coupling with the serine residue of HS-modified protein AGRN, and B4GALT7-EXT1/EXT2 catalyzes the HS chain elongation of AGRN. The HS-modified AGRN protein is transported to the plasma membrane, binds the viral HA protein, and further recruits the endocytic adapter AP2B1 to initiate virus endocytosis. (B) Excessive accumulation of the unmodified AGRN HS mutant due to the deficiency in SLC35B4 expression leads to AP2B1 degradation via the proteasome pathway, thereby inhibiting the endocytosis of IAV.

Taken together, SLC35B4 was responsible for regulating the physiological level of AGRN through transporting UDP-xylose into the HS modification pathway. Upon attachment with SA receptors on the cell surface, the HA protein of IAV binds AGRN, which further recruits endocytic adapter AP2B1 to initiate virus endocytosis (Fig. 10A). Under abnormal circumstances, excessive accumulation of the AGRN HS mutant due to the deficiency of SLC35B4 expression leads to AP2B1 degradation via the proteasome pathway, thereby inhibiting the endocytosis of IAV (Fig. 10B).

## DISCUSSION

Genome-wide siRNA library screens have been widely used to identify host factors involved in IAV replication (43, 44). In the present study, we sought to define the mechanistic basis for the role of SLC35B4, a host factor identified in our previous siRNA library screen, in IAV replication. The replication of different IAV strains was universally inhibited under the condition of SLC35B4 deficiency in A549 cells. Importantly, we found that knockdown of Slc35b4 protein expression obviously reduced virus pathogenicity in *Slc35b4*_KD mice compared with control mice. We observed that the amount of internalized viruses was significantly reduced in *SLC35B4*_KO A549 cells. Due to the defect in internalization caused by SLC35B4 knockout, the subsequent steps in the viral life cycle were impaired, thereby leading to reduced virus replication.

The subcellular localization of host proteins determines, to some extent, the stage in which they play a role during viral infection. However, when IAV entry was stepwise analyzed in *SLC35B4*_KO A549 cells, ER-/Golgi apparatus-resident SLC35B4 unexpectedly regulated the endocytic uptake of viral particles. During IAV entry, the viral HA protein is not only responsible for binding to SA receptors but also hijacks some host factors, such as FFAR2 (15), IGDCC4 (16), and Cav1.2 (45), to accelerate virus endocytosis. However, SLC35B4 did not directly interact with the HA protein. Given that the Golgi apparatus-resident SLC35A1 transports CMP-SA from the cytoplasm into the Golgi apparatus where glycosyltransferases catalyze SA biosynthesis of cell membrane molecules and plays an indirect role in IAV attachment (42), we speculate that SLC35B4 likely utilizes its bifunctional nucleotide sugar (UDP-GlcNAc and UDP-xylose) transporter activity to regulate protein modifications and then indirectly promotes IAV endocytosis.

UDP-GlcNAc (the sugar nucleotide donor for O-GlcNAcylation) is synthesized from glucose through the hexosamine biosynthesis pathway (46). One of the SLC35B4 functions is to transport UDP-GlcNAc from the cytoplasm into the lumen of the ER and/or Golgi apparatus (21). Subsequently, ER-resident EOGT transfers UDP-GlcNAc to Ser/Thr residues in secreted and membrane proteins (23). We found that EOGT knockdown or knockout in A549 cells had no effect on WSN (H1N1) virus replication, demonstrating that SLC35B4-EOGT-mediated O-GlcNAcylation is not involved in IAV endocytosis into host cells. Another function of SLC35B4 is to transport UDP-xylose from the cytoplasm into the lumen of the ER and/or Golgi apparatus (21). The initial transfer of xylose from UDP-xylose to specific Ser residues of proteoglycan core proteins to initiate the biosynthesis of the tetrasaccharide linkage region (GlcAβ1–3Galβ1–3Galβ1–4Xylβ1) is catalyzed by the ER residents XYLT1 and XYLT2 (24). However, in pulmonary epithelial A549 cells, only XYLT2 mRNA is highly expressed according to mRNA transcript analysis (www.proteinatlas.org). ER-resident B4GALT7 (the second rate-limiting enzyme) is involved in the biosynthesis of the tetrasaccharide linkage region by catalyzing the transfer of Gal from the uridine 5'-diphosphogalactose to a xylose residue (39). Glycosyltransferases of the exostosin family, such as Golgi apparatus-residents EXT1 and EXT2, can also catalyze HS chain polymerization on the tetrasaccharide linkage region of HSPGs. In human cells, the HS possesses a negative charge, which interacts electrostatically with basic residues exposed by viral surface glycoproteins. For example, HS chains catalyzed sequentially by XYLT2, B4GALT7, EXT1, and EXT2 have been shown to be an attachment factor for Ebola virus, Marburg virus, Akabane virus, Schmallenberg virus, and SARS-CoV-2 (33, 47, 48). Here, we demonstrated that the siRNA-mediated knockdown of XYLT2, B4GALT7, EXT1, or EXT2 in A549 cells remarkably reduced the growth titers of the WSN (H1N1) virus, indicating that the HS signaling pathway is indeed involved in the regulation of IAV replication.

In mammalian cells, HSPGs are composed of various cell surface or extracellular matrix proteins conjugated with unbranched, negatively charged HS polysaccharides. Of the HSPGs analyzed, we found that AGRN acts as a unique host factor that can promote the replication of different subtypes of IAV. We found that heparinase treatment did not impair IAV infection, and AGRN functioned in IAV internalization rather than attachment, thereby indicating that HS is not an attachment receptor for IAV. Viral entry sometimes requires the interaction of viral envelope proteins with coreceptors or auxiliary factors that are distinct from those mediating initial virus binding. In the present study, regardless of the subtype specificity, AGRN directly interacted with the HA protein of IAV. Furthermore, we found that both the 30–350 region in the N-terminal 110-kDa subunit and the 1325–1548 region in the C-terminal 90-kDa subunit of AGRN mediated the interaction with the HA1 domain of IAV. Although the interactive region (30–350 aa) of AGRN contains two N-linked glycosylation sites (positions 135 and 250), there was no difference in the interaction level of the AGRN 30–350 region with IAV HA1 in normal or *SLC35A1*_KO (deficient in SA expression) HEK293T cells. Thus, such an interaction itself, independent of SA, is important for the role of AGRN in promoting IAV internalization during entry.

AGRN is a secreted extracellular matrix HSPG (41). The mutation of multiple potential HS modification sites of AGRN promoted its intracellular expression and further increased the level of secretory AGRN in the supernatant. Neither secretory wild-type AGRN nor AGRN HS mutant co-incubated with IAV could rescue the virus growth defect in *AGRN*_KO A549 cells. Therefore, there must be other mechanisms to indirectly regulate IAV endocytosis through SLC35B4-mediated HS modification of AGRN. Strikingly, we found that SLC35B4 deficiency in A549 cells led to excessive accumulation of the unmodified AGRN HS mutant and decreased the expression of the endocytic adapter AP2B1. Finally, we revealed that the unmodified HS AGRN mutant was directly involved in the reduction of AP2B1 stability via the proteasomal degradation pathway. Given the importance of AP2B1 in the internalization of IAV (15), our study thus demonstrated that HS-modified ARGN is important in regulating the stability of AP2B1, thereby supporting the replication of IAV.

In summary, here, we demonstrate that IAV endocytosis is independent of the SLC35B4–EOGT-mediated O-GlcNAcation signaling pathway but dependent on the SLC35B4–XYLT2–B4GALT7–EXT1/EXT2-mediated HS-modification pathway in which AGRN acts as an important proviral host factor.

## MATERIALS AND METHODS

### Cells and viruses

Human embryonic kidney cells (HEK293T), human lung carcinoma cells (A549), and Madin–Darby canine kidney (MDCK) cells were cultured at 37°C in a 5% CO_2_ humidified incubator in DMEM (Life Technologies, Grand Island, NY, USA) supplemented with 10% fetal bovine serum (FBS, Sigma-Aldrich, St. Louis, MO, USA), F-12K medium (Life Technologies) supplemented with 10% FBS, and DMEM containing 6% newborn calf serum (Sigma-Aldrich). All media contained 100 U/mL penicillin and 100 µg/mL streptomycin (Life Technologies).

A/WSN/33 (WSN, H1N1), A/Fuzhou/1/2009 (FZ09, H1N1), and A/Anhui/2/2005 (AH05, H5N1) were propagated on MDCK cells cultured in MEM (Life Technologies) supplemented with 0.3% bovine serum albumin (BSA, Sigma Aldrich) and 0.5 µg/mL L-1-tosylamide-2-phenylmethyl chloromethyl ketone (TPCK)-treated trypsin (Worthington, Lakewood, NJ, USA). A/chicken/Shanghai/SC197/2013 (SH13, H9N2) was propagated in embryonated SPF eggs. The H5N1 NA-Venus reporter virus was generated in our laboratory as described previously (35). All experiments with the wild-type AH05 (H5N1) virus or H5N1 NA-Venus reporter virus were conducted within the enhanced animal biosafety level 3 (ABSL3+) facility in the HVRI of CAAS, which is approved for such use by the Ministry of Agriculture and Rural Affairs of China and the China National Accreditation Service for Conformity Assessment.

### Plasmids

The *SLC35B4*, *EOGT*, *OGT*, *B4GALT7*, *EXT1*, *EXT2*, *AGRN*, *Notch1*(1-715), *Notch2*(1-719), *FFAR2, IGDCC4, AP2B1, CLTA, CLTB,* and *CLTC* genes were amplified by RT-PCR from total cellular mRNAs of A549 cells. The *XYLT2*-expressing plasmid was purchased from MiaoLing Plasmid Sharing Platform (Wuhan, China). Open reading frames (ORFs) of *SLC35B4*, *EOGT*, *OGT*, *XYLT2*, *B4GALT7*, *EXT1, EXT2, CLTA, CLTB,* and *CLTC* were cloned into the mammalian expression vector pCAGGS with an Myc or HA tag at the C-terminus. ORFs of *Notch1*(1–715), *Notch2*(1–719), *FFAR2, IGDCC4,* and *AP2B1* genes were inserted into pCAGGS with a V5 tag at the C-terminus. The ORFs of *AGRN* and its truncation mutants or HS mutant (S95/671/674/676/1055/1060/1062/1066/1067/1072/1078/1082/1091/1092/1543A) were cloned into pCAGGS with a Flag tag at the C-terminus. ORFs of the *PB2*, *PB1*, *PA*, *NP*, *HA*, *NA, M1*, and *M2* of the WSN (H1N1) virus were cloned into pCAGGS. The *HA1* and *HA2* of the WSN (H1N1) virus and the *HA1* of AH05 (H5N1), SH13 (H9N2), and FZ09 (H1N1) virus were cloned into pCAGGS with an Myc tag at the C-terminus. All the constructs were verified by sequencing.

### Antibodies

The commercially obtained primary antibodies used in this study were as follows: mouse anti-Flag monoclonal antibody (mAb) (A00187-100, GenScript, Nanjing, China), mouse anti-HA mAb (A01244-100, GenScript), mouse anti-Myc mAb (M4439, Sigma-Aldrich), mouse anti-GAPDH mAb (60004-1-Ig, Proteintech, Wuhan, China), mouse anti-O-GlcNAc (CTD110.6) mAb (9875S, Cell Signaling, Danvers, MA, USA), mouse anti-M1 mAb (ab22396, Abcam, Cambridge, MA, USA), rabbit anti-Myc polyclonal antibody (pAb) (A00172-40, GenScript), rabbit anti-V5 pAb (AB3792, Millipore, Darmstadt, Germany), rabbit anti-GAPDH pAb (10494-1-AP, Proteintech), rabbit anti-AGRN pAb (PA5-109369, Life Technologies), rabbit anti-MX1 pAb (13750-1-AP, Proteintech), rabbit anti-CANX pAb (ab22595, Abcam), rabbit anti-GM130 pAb (11308-1-AP, Proteintech), rabbit anti-EOGT pAb (A21166, Abclonal, Woburn, MA, USA), rabbit anti-CLTA pAb (A3793, Abclonal), rabbit anti-CLTB pAb (A8404, Abclonal), rabbit anti-CLTC pAb (A12423, Abclonal), rabbit anti-AP2B1 pAb (ab220778, Abcam), rabbit anti-SLC35B4 pAb (orb155294, Biorbyt, Cambridge, United Kingdom), rabbit anti-SLC35B4 pAb (83527-3-RR, Proteintech), rabbit anti-XYLT2 pAb (ab155193, Abcam), rabbit anti-EXT1 pAb (A2030, Abclonal), rabbit anti-EXT2 pAb (GTX101934, Genetex, Irvine, CA, USA), rabbit anti-HA (IAV) pAb (11692-T54, Sino Biological, Beijing, China), rabbit anti-NA pAb (GTX629696, GeneTex), rabbit anti-M1 pAb (GTX125928, GeneTex), rabbit anti-M2 pAb (GTX125951, GeneTex), and rabbit anti-NS1 pAb (GTX633686, GeneTex). The mouse anti-PB2 mAb, mouse anti-PB1 mAb, mouse anti-PA mAb, mouse anti-NP mAb, and rabbit anti-NP pAb were prepared and stored in our laboratory (49). Alexa Fluor 488 goat anti-mouse IgG (H + L) (A11029), Alexa Fluor 488 goat anti-rabbit IgG (H + L) (A11008), Alexa Fluor 633 goat anti-mouse IgG (H + L) (A21050), and Alexa Fluor 633 goat anti-rabbit IgG (H + L) (A21071), obtained from Life Technologies, were used as secondary antibodies for confocal microscopy. The secondary antibodies used for Western blotting were DyLight 800 goat anti-rabbit IgG (H + L) and DyLight 700 goat anti-mouse IgG (H + L) (Immunoway, Plano, TX, USA).

### Impact of siRNA knockdown of SLC35B4 on the replication of the H5N1 NA-Venus reporter virus

A549 cells seeded in 384-well plates were transfected with *SLC35B4* siRNA-1 (5′-UCAUGAACAUCAUCACUCA-3′), *SLC35B4* siRNA-2 (5′-GCAAAUUUGUGAGCCUCAU-3′), or scrambled siRNA (5′-UUCUUCCGAACGUGUCACGU-3′) (GenePharma, Shanghai, China) at a concentration of 30 nM by using the Lipofectamine RNAiMAX Transfection Reagent (Invitrogen, Carlsbad, CA, USA). At 48 hours post-transfection, knockdown of SLC35B4 was verified by RT-qPCR. siRNA-treated A549 cells were infected for 24 hours with H5N1 NA-Venus virus (MOI = 0.1), fixed with 4% paraformaldehyde (PFA, Solarbio, Beijing, China) for 30 minutes, and stained with Hoechst 33342 for 30 minutes at room temperature to label their nuclei. Images were captured by using the Operetta high-content imaging system (PerkinElmer, Waltham, MA, USA), and the cell infection ratio was calculated according to the Venus fluorescence intensity. The inhibitory effect of siRNA on virus replication is shown as a relative ratio, which was calculated by dividing the average fluorescence intensity of three cell wells containing *SLC35B4* siRNA with that of three wells containing scrambled siRNA.

### Replication evaluation of wild-type IAVs or VSV-EGFP in SLC35B4- or associated gene-knockdown A549 cells

SiRNA targeting *SLC35B4*, *EOGT* (5′-GCGAACCUCUGUAUAACUA-3′), *XYLT2* (5′-CCAGCAGGAGAUCGCCAAU-3′ or 5′-GGAUGUACCUGCGGAGCAU-3′), *B4GALT7* (5′-GCAACAGCACGGACUACAU-3′ or 5′-GCUGGACUAUGGCUUUCCUT-3′), *EXT1* (5′-GCUUCAAAGUCUACGUAUA-3′ or 5′-GCCAAAGCCAGCAUCAGUA-3′), *EXT2* (5′-CCCUCAUCCCAAGAAUGAA-3′ or 5′-CCAUCUCCCGGGAGUAUAA-3′), *AGRN* (5′-GCCUGCAAAUCUCUAUCCA-3′ and 5′-CCUUUGUCGAGUACCUCAA-3′), *SDC1* (5′-GCAAAUUGUGGCUACUAAU-3′ and 5′-GGAGAAUACGGCUGUAGUG-3′), *GPC1* (5′-CCCUGACUAUUGCCGAAAU-3′ and 5′-GGACACUGUGCAGUGAGAA-3′), *NRP1* (5′-AACACCUAGUGGAGUGAUAAA-3′ and 5′-AACAGCCUUGAAUGCACUUAU-3′), *TGFBR3* (5′-GCUUUCCAGGUGGAUAUAA-3′ and 5′-GCUUUGGACAAUGGCUAUA-3′), or *HSPG2* (5′-GCGGCUCCCUGCGUUACAA-3′ and 5′-GCACCUUCAUUGUGCCUUU-3′), or scrambled siRNA at a concentration of 30 nM was transfected into A549 cells seeded in 12-well plates by using the Lipofectamine RNAiMAX Transfection Reagent. At 48 hours post-transfection, the siRNA-treated A549 cells were infected with WSN (H1N1) (MOI = 0.01), AH05 (H5N1) (MOI = 0.1), SH13 (H9N2) (MOI = 0.1), or FZ09 (H1N1) (MOI = 0.1) viruses. Supernatants were collected at 24 h p.i. and 48 h p.i., and virus titers were determined by performing plaque assays on MDCK cells. In a separate experiment, A549 cells treated with *SLC35B4* siRNA-1, *SLC35B4* siRNA-2, or scrambled siRNA were infected with VSV-EGFP (100 50% tissue culture infective doses [TCID_50_]). Supernatants were collected at 12 h p.i. and titrated on MDCK cells by calculating the TCID_50_.

### Generation of *SLC35A1*_KO, *SLC35B4*_KO, *EOGT*_KO, and *AGRN*_KO A549 cells and virus infection

Single-guide RNA (sgRNA) was designed by using the Zhangfeng lab website. The sgRNA sequences targeting the indicated genes were as follows: 5′-TTCTGTGATACACACGGCTG-3′ and 5′-GCTGGCGTCTACTTGTCAGA-3′ (*SLC35A1*); 5′-GCGGTGGGCCTGGTGTTCGC-3′ and 5′-CATGTTCTTCACCGTGAGCG-3′ (*SLC35B4*), 5′-GAATGTGCTCCTCTGGCAAG-3′ and 5′-GGACACTCTTGAGTCAGCTG-3′ (*EOGT*), 5′-GCACACGGCATTGAACCGGC-3′ and 5′-GATCCTGTGTGCGGCAGCGA-3′ (*AGRN*-1), and 5′-GGTCGTAGCGGAACTCCAGG-3′ (*AGRN*-2). The sgRNA sequences were cloned into the pSpCas9(BB)−2A-GFP (pX458) vector (50), which also contains an expression cassette for Cas9 and EGFP. The pX458 constructs (2 µg) targeting a given gene were simultaneously electrotransfected into A549 cells by using the Neon Transfection System (Thermo Fisher Scientific, Waltham, MA, USA). The electrotransfected cells were trypsinized 48 hours later, and the single cells were then seeded into each well of a 96-well plate by using a SONY-MA900 Flow Cell Sorter (Sony, Japan). The knockout of gene expression was confirmed by PCR, sequencing, or Western blotting. *SLC35B4*_KO, *EOGT*_KO, *AGRN*_KO, or control A549 cells were infected with WSN (H1N1) (MOI = 0.01), AH05 (H5N1) (MOI = 0.1), SH13 (H9N2) (MOI = 0.1) or FZ09 (H1N1) (MOI = 0.1) viruses. Supernatants were collected at 24 h p.i. and 48 h p.i., and virus titers were determined by using plaque assays on MDCK cells. In a separate experiment, *SLC35B4*_KO and control A549 cells were infected with VSV-EGFP (100 TCID_50_). Supernatants were collected at 12 h p.i. and titrated on MDCK cells by calculating the TCID_50_.

### Viral pathogenicity in *Slc35b4_*KD and control mice

To determine the effect of SLC35B4 on the pathogenicity of IAV *in vivo*, two 2′-O-methoxylated (2′-Ome) and 3′-cholesterol (3′chol)-conjugated *Slc35b4* siRNAs (5′-GCCACCAGCUAUCCCAAUA-3′ and 5′-GCACCUCGUUUGUCUUCAU-3′, GenePharma) were intratracheally injected into eight 6-week-old female C57BL/6J mice (5 nmol for each siRNA). After two injections at a 36-hour interval, three *Slc35b4*_KD mice or control mice treated with 2′-Ome and 3’chol-conjugated scrambled siRNA (5’-UUCUCCGAACGUGUCACGU-3’) were euthanized, and their lungs were homogenized for Western blotting with an anti-SLC35B4 pAb. Meanwhile, five *Slc35b4_*KD or control mice were inoculated intranasally with 5 MLD_50_ of the WSN (H1N1) virus, monitored daily for 14 days for body weight loss and mortality, and were humanely euthanized in the case of decrease in over 70% of their initial body weight or at the end of the 14-day observation period.

### Analysis of gene downregulation efficiency and level of viral RNA species

A549 cells seeded in 12-well plates were transfected with siRNA targeting *SLC35B4*, *EOGT*, *XYLT2*, *B4GALT7*, *EXT1*, *EXT2*, *AGRN*, *SDC1*, *GPC1*, *NRP1*, *TGFBR3,* or *HSPG2* or with scrambled siRNA at a concentration of 30 nM by using the Lipofectamine RNAiMAX Transfection Reagent. At 48 hours post-transfection, total RNA was extracted by using the RNeasy Plus Mini Kit (QIAGEN, Valencia, CA, USA) according to the manufacturer’s instructions. The first-strand cDNAs were synthesized with the oligo(dT) primer and random 6 mers by using HiScript II qRT SuperMix for qPCR (+gDNA wiper) (Vazyme, Nanjing, China). RT-qPCR assays were performed by using ChamQ Universal SYBR qPCR Master Mix (Vazyme). Relative RNA quantities were determined by using the comparative cycle-threshold method, with cellular *GAPDH* serving as the internal control. Dissociation curve analysis was performed after each assay to ensure specific detection.

The *SLC35B4*_KO or control A549 cells grown in 12-well plates were infected with the WSN (H1N1) virus (MOI = 5). Total RNA was extracted at 4 h p.i. and 6 h p.i. by using TRIzol Reagent (Life Technologies) according to the manufacturer’s instructions. Relative quantities of viral NP vRNA, cRNA, and mRNA were determined by RT-qPCR as described previously (51), with *GAPDH* serving as the endogenous reference.

### Effect of SLC35B4 or AGRN knockout on the expression of IAV proteins

The *SLC35B4*_KO, *AGRN*_KO, or control A549 cells were seeded in 12-well plates to 90% confluence and then infected with the WSN (H1N1) virus (MOI = 5). At 3 h p.i., 6 h p.i., and 9 h p.i., cell lysates were prepared with SDS-PAGE loading buffer (Solarbio) and then subjected to Western blotting with a mouse anti-PB2/PB1/PA mAb and a rabbit anti-NP/HA/NA/NS1/M1/M2 pAb to determine the levels of viral proteins.

### Confocal microscopy to visualize NP accumulation in the nucleus of infected cells

*SLC35B4*_KO, *AGRN*_KO, or control A549 cells were seeded in glass-bottom dishes to 90% confluence and then infected with the WSN (H1N1) virus (MOI = 5). At the indicated time points p.i., cells were fixed with 4% PFA for 30 minutes and permeabilized with 0.5% Triton X-100 in PBS for 15 minutes. The permeabilized cells were blocked with 5% BSA in PBS for 1 hour and incubated with mouse anti-NP mAb (1:200) at 4°C overnight. The cells were then washed three times with PBS and incubated with Alexa Fluor 633 goat anti-mouse IgG (H+L) for 1 hour at room temperature. After three washes, the cells were incubated with 4′,6-diamidino-2-phenylindole (DAPI; Thermo Fisher Scientific) for 15 minutes to stain the nuclei. Images were acquired by using an LSM 800 confocal microscope with Airyscan (Zeiss, Oberkochen, Germany).

### Subcellular distribution and colocalization detection

A549 cells seeded in glass-bottom dishes (90% confluence) were transiently transfected with plasmids expressing SLC35B4-Myc, EOGT-Myc, OGT-Myc, XYLT2-Myc, or B4GALT7-Myc; co-transfected with plasmids expressing SLC35B4-Myc and EOGT-HA, XYLT2-HA, B4GALT7-HA, or EXT1-HA/EXT2-HA; co-transfected with plasmids expressing EXT1-Myc and EXT2-HA; or co-transfected with plasmids expressing AGRN-Flag and AP2B1-V5 by using Lipofectamine 3000 Transfection Reagents (Invitrogen). At 24 hours post-transfection, the cells were washed with PBS, fixed with 4% PFA for 30 minutes, and permeabilized with 0.5% Triton X-100 in PBS for 15 minutes. The permeabilized cells were blocked with 5% BSA in PBS for 1 hour and then incubated with primary antibodies at 4°C overnight. The cells were washed three times with PBS and incubated with Alexa Fluor-conjugated secondary antibodies for 1 hour at room temperature. After three washes, the cells were incubated with DAPI for 15 minutes to stain the nuclei. Images were acquired by using an LSM 800 confocal microscope with Airyscan.

### Generation of *SLC35B4*_KO HEK293T cells and dual-luciferase reporter assay

Two sgRNAs (2 µg) targeting SLC35B4 were simultaneously transfected into HEK293T cells by using Lipofectamine LTX and Plus Reagents (Invitrogen). At 36 hours post-transfection, the cells were trypsinized, and the single cells were then seeded into each well of a 96-well plate by using a SONY-MA900 Flow Cell Sorter (Sony). Knockout of SLC35B4 gene expression was confirmed by PCR and sequencing. *SLC35B4*_KO or control HEK293T cells were co-transfected with the four vRNP protein expression plasmids of the WSN (H1N1) virus (PB2, PB1, PA, and NP, 0.2 µg of each), the construct pHH21-SC09NS F-Luc (0.2 µg), and an internal control pRL-TK (0.01 µg) by using Lipofectamine 3000 transfection reagents. At 36 hours post-transfection, the cells were split by using the dual-luciferase reporter assay system (Promega, Madison, WI, USA), and the luciferase activities were measured on a GloMax 96 Microplate Luminometer (Promega).

### Cell viability assay

Cell viability was determined by using the CellTiter-Glo kit (Promega) as described previously (52, 53). Briefly, *SLC35A1*_KO, *SLC35B4*_KO, or control HEK293T cells and *SLC35B4*_KO, *EOGT*_KO, *AGRN*_KO, or control A549 cells seeded in opaque-walled 96-well plates were cultured for 24 hours; A549 cells transfected with siRNA targeting *SLC35B4*, *EOGT*, *XYLT2*, *B4GALT7*, *EXT1*, *EXT2*, *AGRN*, *SDC1*, *GPC1*, *NRP1*, *TGFBR3*, or *HSPG2* or scrambled siRNA at a concentration of 30 nM in opaque-walled 96-well plates were cultured for 48 hours. CellTiter-Glo reagent (100 µL) was then added directly into each well to induce cell lysis on a shaker for 10 minutes, and the luminescence of the cell lysates was then measured with the GloMax 96 Microplate Luminometer.

### MX1 expression under IFN-α or IFN-β stimulation

A549 cells grown in 12-well plates were transfected with *SLC35B4* siRNA or scrambled siRNA (30 nM) for 48 hours and then left untreated or treated with 100 U/mL of IFN-α (Sigma-Aldrich) or 25 pg/mL of IFN-β (R&D Systems, Minneapolis, MN, USA) for 24 hours. Cell lysates were then subjected to Western blotting with a rabbit anti-MX1 pAb to determine the level of MX1 protein expression.

### Analysis of sialic acid (SA) receptors

*SLC35A1*_KO or control HEK293T cells and *SLC35A1*_KO, *SLC35B4*_KO, *AGRN*_KO, or control A549 cells were seeded in 6-well plates to 90% confluence. The cells were trypsinized, fixed with 4% PFA for 20 minutes, and then stained with the Alexa Fluor 633 conjugate of wheat germ agglutinin (WGA; an agent for total SA detection) (Invitrogen). After three washes with PBS, the cell suspensions were subjected to flow cytometry on a FACSAria flow cytometer (BD Biosciences, Franklin Lakes, NJ, USA). In a separate experiment, *SLC35A1*_KO, *SLC35B4*_KO, or control A549 cells were also stained with biotinylated *Maackia amurensis* lectin (MAL) (α-2,3-SA) or *Sambucus nigra* lectin (SNA) (α-2,6-SA) (Vector Laboratories, Burlingame, CA, USA). The bound biotinylated lectins were captured by streptavidin conjugated with Cy5 fluorophore (Life Technologies). After three washes, the cell suspensions were subjected to flow cytometry on the FACSAria flow cytometer.

### Virus attachment assay

*SLC35A1*_KO, *SLC35B4*_KO, *AGRN*_KO, or control A549 cells were grown in six-well plates to 90% confluence and then infected with the WSN (H1N1) virus (MOI = 5) on ice at 4°C for 1 hour. The cells were trypsinized, fixed with 4% PFA for 30 minutes, and then stained with the primary rabbit anti-HA pAb and secondary Alexa Fluor 488 goat anti-rabbit IgG (H+L) antibody. After five washes with PBS, the cell suspensions were subjected to flow cytometry on the FACSAria flow cytometer. The data were analyzed by using FlowJo software.

### Virus internalization assay

*SLC35B4*_KO, *AGRN*_KO, and control A549 cells grown in glass-bottom dishes were infected with the WSN (H1N1) virus (MOI = 5) on ice at 4°C for 1 hour, and then the temperature was shifted to 37°C to allow internalization. The cells were fixed with 4% PFA at the indicated time points post temperature shift (p.t.s.) and then stained with the rabbit anti-HA pAb and HRP-labeled goat anti-rabbit IgG antibody according to the tyramine signal amplification (TSA) protocol (APExBIO, Houston, TX, USA). The plasma membrane and intracellular membrane structures were stained with CellBrite Cytoplasmic Membrane Dyes. The cells were incubated with DAPI for 15 minutes to stain the nuclei. Images were acquired by using the LSM 800 confocal microscope with Airyscan.

*SLC35B4*_KO and control A549 cells were grown in six-well plates to 90% confluence and then infected with the WSN (H1N1) virus (MOI = 5) for 1 hour on ice at 4°C, followed by a temperature shift to 37°C for 10 minutes and 20 minutes to allow virus internalization. The cells were then washed five times with ice-cold PBS-HCl (pH 1.3) to remove the attached but not-yet-internalized virions. Total RNA was then extracted by using TRIzol reagent (Life Technologies) according to the manufacturer’s instructions. Relative quantities of viral NP genomic RNA (vRNA) were determined by RT-qPCR as described previously (51), with 16 s RNA serving as the endogenous reference.

*SLC35A1*_KO, *SLC35B4*_KO, *AGRN*_KO, or control A549 cells grown in six-well plates to 90% confluence were infected with the WSN (H1N1) virus (MOI = 10) for 1 hour on ice at 4°C, followed by a culture temperature shift to 37°C for 30 minutes to allow internalization. The cells were then washed five times with ice-cold PBS-HCl (pH 1.3) to remove the attached but not-yet-internalized virions. Then, the cells were lysed with SDS-PAGE loading buffer (Solarbio) and subjected to Western blotting with a rabbit anti-NP pAb and a rabbit anti-GAPDH pAb.

### Fusion/uncoating assay

*SLC35B4*_KO and control A549 cells were infected with the WSN (H1N1) virus (MOI = 10) for 1 hour on ice at 4°C. After removing the inoculum and supplementing with the fresh medium, the culture temperature was shifted to 37°C. The cells were fixed at 0 hours and 1 hour post-temperature shift and permeabilized with 0.5% Triton X-100 in PBS for 15 minutes. The permeabilized cells were blocked with 5% BSA in PBS for 1 hour and then incubated with a mouse anti-M1 mAb at 4°C overnight. The cells were washed three times with PBS and incubated with Alexa Fluor 488 goat anti-mouse IgG (H+L) for 1 hour. After three washes, the cells were incubated with DAPI for 15 minutes to stain the nuclei. Images were acquired by using the LSM 800 confocal microscope with Airyscan. A549 cells pretreated with bafilomycin A1 (10 nM) for 1 hour and further infected with the WSN (H1N1) virus were included as a positive control.

### Lysotracker staining

*SLC35B4*_KO or control A549 cells were grown in six-well plates to 90% confluence and then pretreated with DMSO or 10 nM bafilomycin A1 for 1 hour. Lysotracker red DND-99 (L7528, Life Technologies) was diluted in F-12K medium containing 0.3% BSA and added to cells at a final concentration of 25 nM. The cells were incubated at 37°C for 30 minutes and then washed three times with the medium. The samples were subjected to flow cytometry on the FACSAria flow cytometer or the LSM 800 confocal microscope with Airyscan.

### Co-immunoprecipitation assay

The indicated plasmids were individually transfected or co-transfected into HEK293T cells grown in six-well plates by using the Lipofectamine LTX and Plus Reagents. At 36 hours post-transfection, the cells were washed once with ice-cold PBS (pH 7.2) and lysed with IP lysis buffer (25 mM Tris-HCl [pH 7.4], 150 mM NaCl, 1% NP-40, 1 mM EDTA, and 5% glycerol) (Pierce, Rockford, IL, USA) containing a complete protease inhibitor cocktail (Roche Diagnostics GmbH, Mannheim, Germany) and PMSF (Beyotime, Shanghai, China) for 1 hour on ice. The supernatants were then immunoprecipitated with primary antibody and protein A/G-agarose (Abmart, Berkeley Heights, NJ, USA) and shaken at 4°C overnight. The beads were then washed twice with ice-cold PBS (pH 7.2), and the bound proteins were separated by SDS-PAGE and detected by Western blotting.

### Western blotting

Protein samples fractionated by SDS-PAGE were transferred onto nitrocellulose membranes (GE Healthcare, Pittsburgh, PA, USA) by using the eBLOT L1 transfer system (GenScript). Membranes blocked with 5% skim milk (TSINGKE, Beijing, China) in PBS were incubated overnight at 4°C with the appropriately diluted primary antibody in PBS containing 0.5% BSA. After incubation with DyLight 800 goat anti-rabbit IgG (H+L) or DyLight 700 goat anti-mouse IgG (H+L), blots were visualized by using an Odyssey CLX infrared imaging system (Li-Cor BioSciences, Lincoln, NE, USA).

### O-GlcNAcylation detection

Plasmids expressing SLC35B4-Myc or EOGT-Myc were individually transfected or co-transfected with plasmids expressing Notch1 (1–715)-V5, Notch2 (1–719)-V5, IGDCC4-V5, or FFAR2-V5 into HEK293T cells in six-well plates by using the Lipofectamine LTX and Plus Reagents. At 36 hours post-transfection, the cells were lysed with IP lysis buffer. The supernatants were immunoprecipitated with a rabbit anti-V5 pAb and protein A/G-agarose (Abmart) and rocked at 4°C overnight. The beads were then washed three times with ice-cold PBS (pH 7.2), and the bound proteins were separated by SDS-PAGE and detected by Western blotting with a mouse anti-V5 mAb and a mouse anti-O-GlcNAc (CTD110.6) mAb to reveal the immunoprecipitated protein and O-GlcNAc-modified protein.

### Heparinase treatment and virus infection

A549 cells were grown in 12-well plates to 90% confluence and then incubated with/without heparinase II (4U) in F-12K medium containing 2 mM CaCl_2_ for 1 hour at 37°C. The cells were infected with the WSN (H1N1) virus (MOI = 0.01) for 1 hour at 4°C. After two washes with F-12K medium, the cells were incubated with F-12K medium containing 0.3% BSA and 0.5 µg/mL TPCK-treated trypsin at 37°C in a 5% CO_2_ humidified incubator. Supernatants were collected at 24 h p.i., and virus titers were determined by plaque assays on MDCK cells.

### Statistical analysis

Statistical significance was determined by using an analysis of variance (ANOVA) (Fig. S7H) or a two-tailed unpaired Student’s *t*-test (the remaining figures) with GraphPad Prism 8 software (GraphPad, San Diego, CA, USA). *P* values of <0.05 were considered significant.

## Data Availability

All relevant data are within the manuscript and its supplemental files.

## SUPPLEMENTAL MATERIAL

Supplemental figures mbio.00194-25-s0001.doc Figures S1 to S11.

ASM does not own the copyrights to Supplemental Material that may be linked to, or accessed through, an article. The authors have granted ASM a non-exclusive, world-wide license to publish the Supplemental Material files. Please contact the corresponding author directly for reuse.
