# Supplementary material for: Cellular SLC35B4 promotes internalization during influenza A virus entry
Source: mBio. 2025 Mar 25;16(5):e00194-25. doi: 10.1128/mbio.00194-25 (PMC12077083; doi:10.1128/mbio.00194-25)
Supplement: Supplemental figures — Figures S1 to S11. [file mbio.00194-25-s0001.doc]

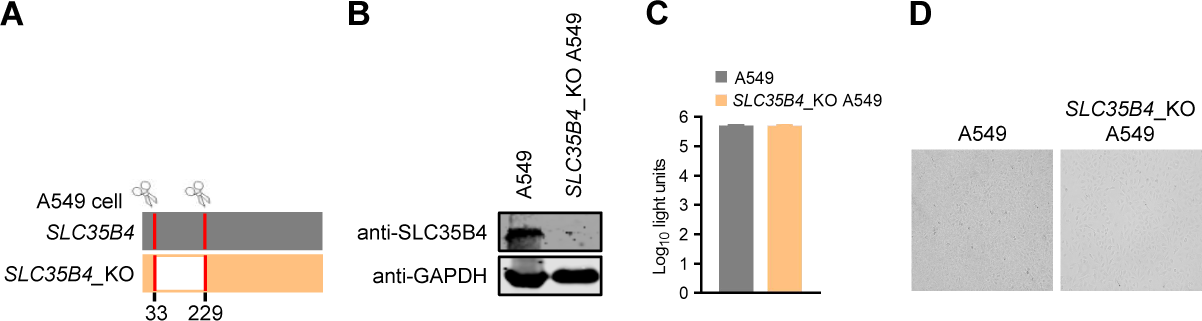


**Figure S1 Generation of *SLC35B4*_KO A549 cells.** (A) Schematic diagram of two sgRNA targeting sites at the *SLC35B4* gene loci and the corresponding truncated mutant validated by sequencing. (B) Knockout of SLC35B4 in *SLC35B4*_KO A549 cells confirmed by western blotting with a rabbit anti-SLC35B4 pAb. (C) Viability of *SLC35B4*_KO A549 cells (n = 3 biologically independent samples). (D) Cellular morphology of *SLC35B4*_KO A549 cells.

**
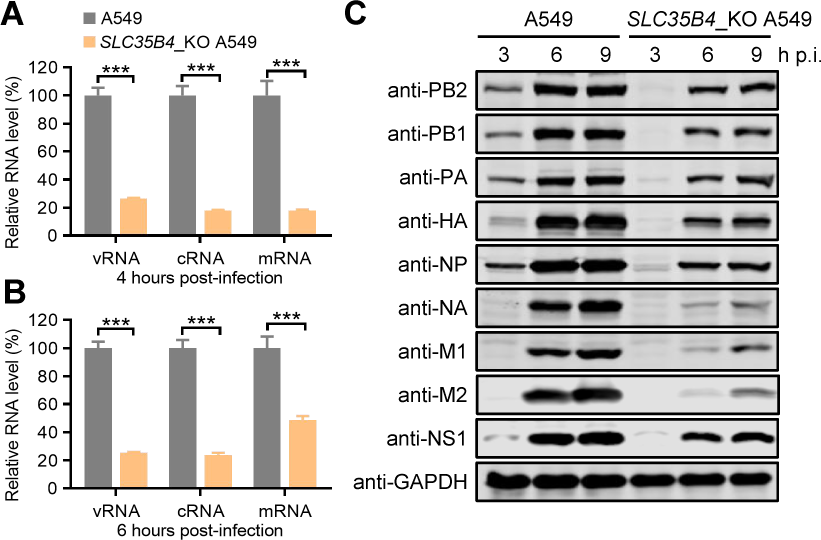
**

**Figure S2 SLC35B4 knockout reduces the levels of viral NP RNAs and different viral proteins.** (A and B) *SLC35B4*_KO or control A549 cells were infected with WSN (H1N1) virus at an MOI of 5. The levels of NP-specific vRNA, cRNA and mRNA were analyzed by RT-qPCR at 4 h p.i. (A) and 6 h p.i. (B), and normalized to *GAPDH* mRNA. The values shown are standardized to the corresponding RNA expression level in control A549 cells (n = 3 biologically independent samples). ***, *P* < 0.001. (C) *SLC35B4*_KO or control A549 cells were infected as in (A) and (B), and the levels of different viral proteins were detected by western blotting at the indicated timepoints p.i.

**
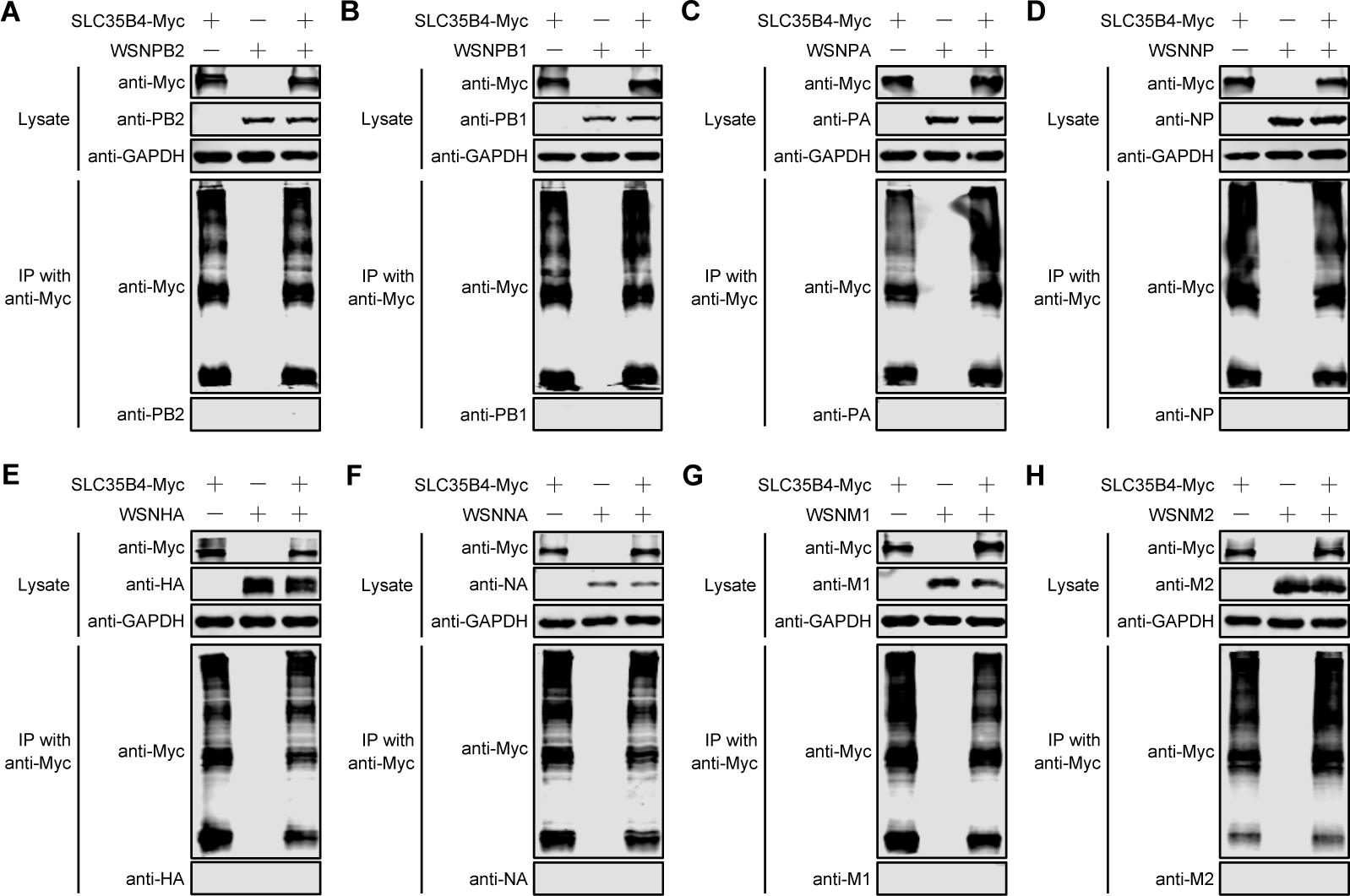
**

**Figure S3 SLC35B4 does not interact with the viral proteins of IAV.** (A to H) Plasmids expressing SLC35B4-Myc and PB2 (A), PB1 (B), PA (C), NP (D), HA (E), NA (F), M1 (G) or M2 (H) of WSN (H1N1) virus were transfected individually or co-transfected into HEK293T cells. At 36 hours post-transfection, cell lysates were immunoprecipitated with a mouse anti-Myc mAb, and then subjected to western blotting with the indicated antibodies.

**
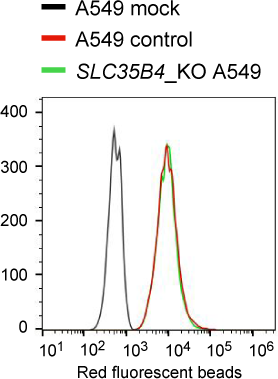
**

**Figure S4 SLC35B4 knockout has no effect on the general endocytosis of A549 cells.** *SLC35B4*_KO and control A549 cells were incubated with red fluorescent beads (1 µM diameter) for 30 minutes at 37℃. The phagocytosis was stopped by adding ice-cold PBS, and the cells were trypsinized and fixed. The cell suspensions were then subjected to flow cytometry.


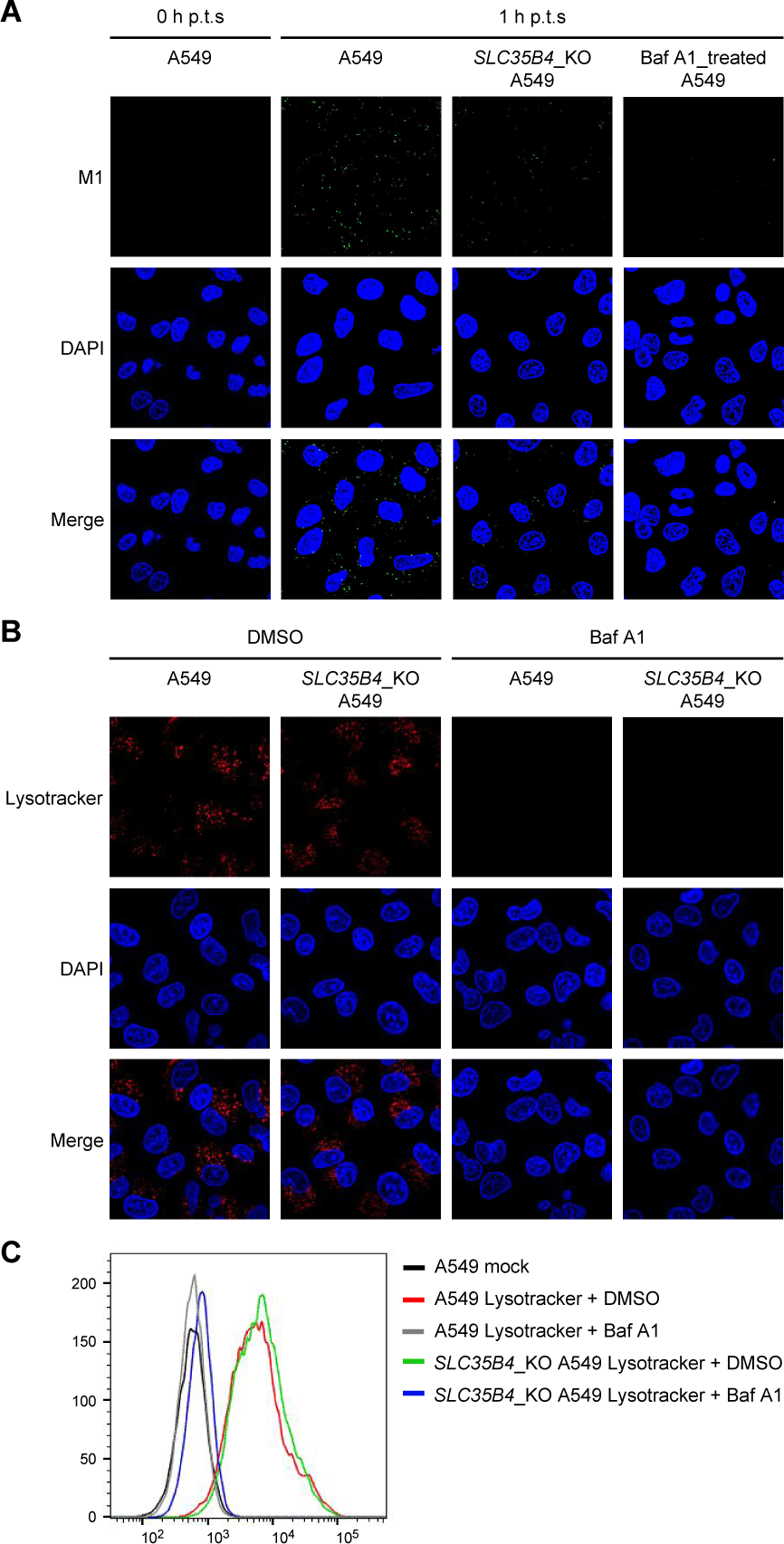


**Figure S5 SLC35B4 engages in a process prior to viral fusion/uncoating.** (A) *SLC35B4*_KO, Bafilomycin A1 (Baf A1)-treated or control A549 cells were infected with WSN (H1N1) virus (MOI = 10) at 4 ℃ for 1 hour, and then shifted to 37℃ for 0 or 1 hour. The cells were fixed and stained with a mouse anti-M1 mAb, followed by incubation with Alexa Fluor 488 goat anti-mouse IgG (H+L) (green). (B and C) *SLC35B4*_KO or control A549 cells were treated with DMSO or Baf A1 for 1 hour, and then labeled with Lysotracker (red) for 30 minutes. The samples were analyzed by confocal microscopy (B) or flow cytometry (C).

**
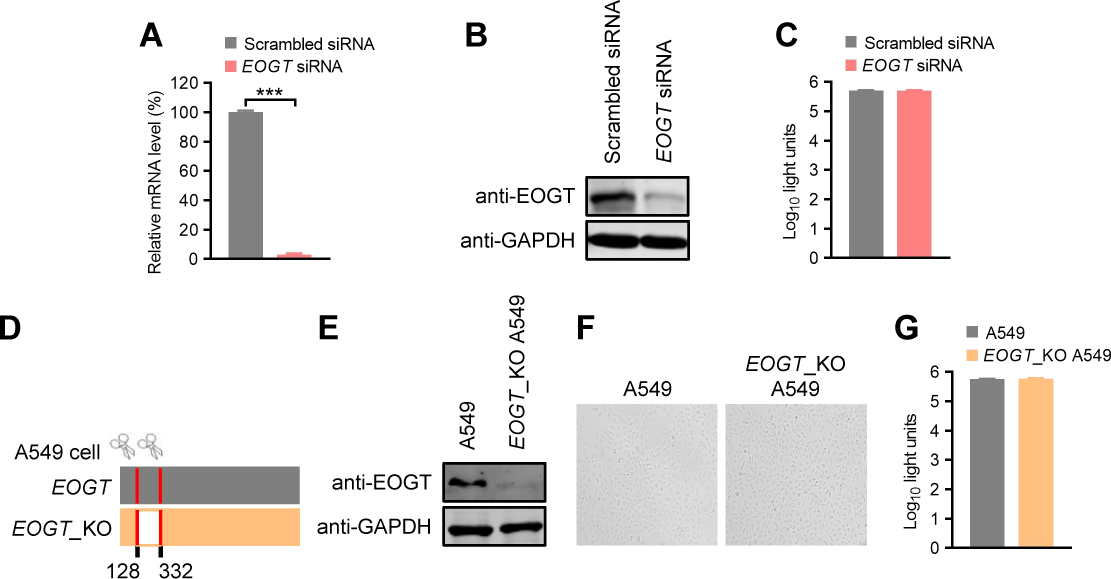
**

**Figure S6 Phenotypes of EOGT knockdown or knockout cells.** (A) *EOGT* mRNA level in *EOGT* siRNA-treated A549 cells was analyzed by RT-qPCR (n = 3 biologically independent samples). ***, *P* < 0.001. (B) EOGT protein level in *EOGT* siRNA-treated A549 cells was evaluated by western blotting with a rabbit anti-EOGT pAb. (C) Viability of A549 cells transfected with *EOGT* siRNA or scrambled siRNA for 48 hours was analyzed by using a CellTiter-Glo assay (n = 3 biologically independent samples). (D) Schematic diagram of two sgRNA targeting sites at the *EOGT* gene loci and the corresponding truncated mutant validated by sequencing. (E) Knockout of EOGT expression in *EOGT*_KO A549 cells was confirmed by western blotting with a rabbit anti-EOGT pAb. (F) Cellular morphology of *EOGT*_KO A549 cells. (G) Viability of *EOGT*_KO A549 cells was analyzed by using a CellTiter-Glo assay (n = 3 biologically independent samples).

**
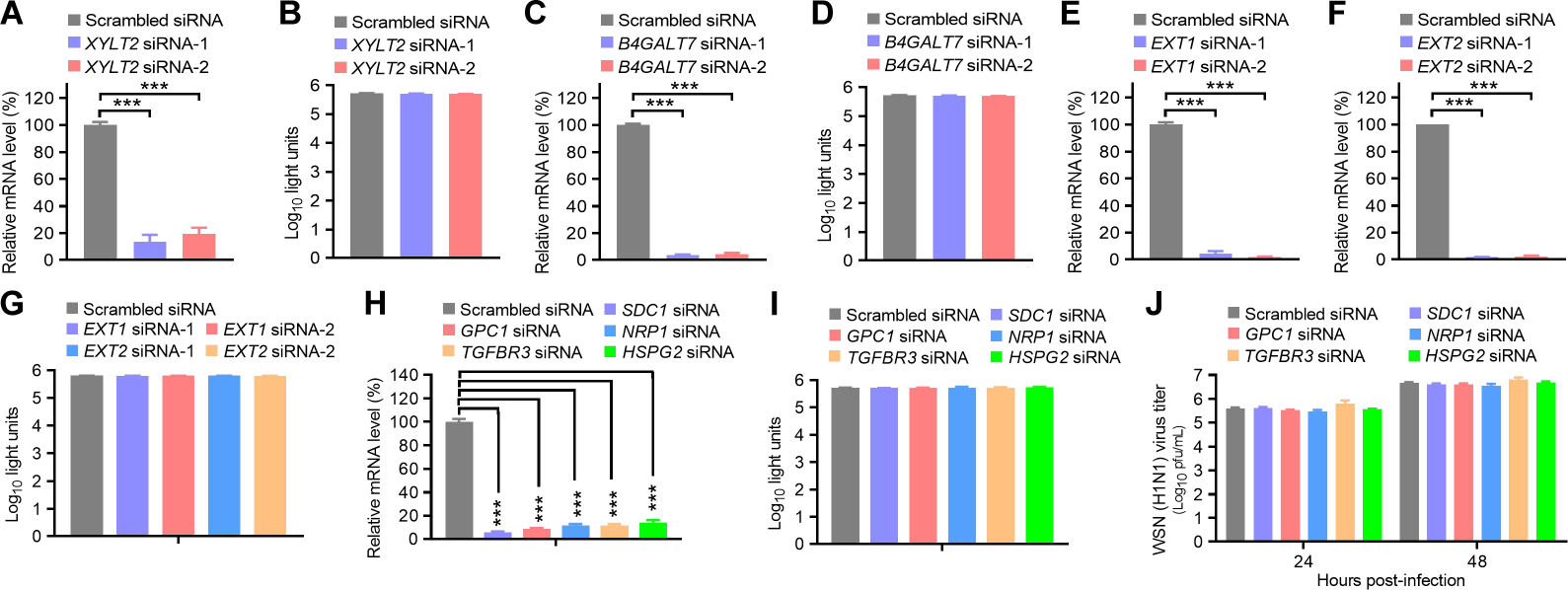
**

**Figure S7 Phenotypes of A549 cells treated with siRNAs targeting O-xylose modification associated factors.** (A, C, E, F) mRNA level of *XYLT2* (A), *B4GALT7* (C), *EXT1* (E) or *EXT2* (F) in A549 cells transfected with gene-specific siRNA or scrambled siRNA for 48 hours (n = 3 biologically independent samples). ***, *P* < 0.001. (B, D, G) Viability of A549 cells transfected with *XYLT2* siRNA (B), *B4GALT7* siRNA (D), *EXT1* siRNA or *EXT2* siRNA (G) for 48 hours was analyzed by using a CellTiter-Glo assay (n = 3 biologically independent samples). (H) mRNA level of *SDC1*, *GPC1*, *NRP1*, *TGFBR3* or *HSPG2* in A549 cells transfected with gene-specific siRNA or scrambled siRNA for 48 hours. ***, *P* < 0.001. (I) Viability of A549 cells transfected with specific siRNA targeting *SDC1*, *GPC1*, *NRP1*, *TGFBR3* or *HSPG2,* or scrambled siRNA for 48 hours was analyzed by using a CellTiter-Glo assay (n = 3 biologically independent samples). (J) A549 cells treated with siRNA targeting *SDC1*, *GPC1*, *NRP1*, *TGFBR3* or *HSPG2* or with scrambled siRNA were infected with WSN (H1N1) virus (MOI = 0.01), and virus titers were determined by means of plaque assays at the indicated timepoints (n = 3 biologically independent samples).

**
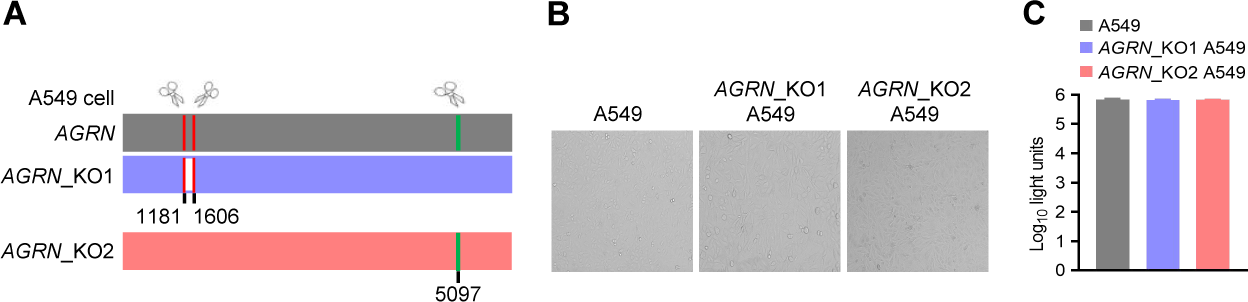
**

**Figure S8** **Generation of *AGRN*_KO A549 cells.** (A) Schematic diagram of targeting sites of a pair of gRNAs or a single gRNA at the *AGRN* gene loci and the corresponding truncated mutants validated by sequencing. (B) Cellular morphology of *AGRN*_KO A549 cells. (C) Viability of *AGRN*_KO A549 cells was analyzed by using a CellTiter-Glo assay (n = 3 biologically independent samples).


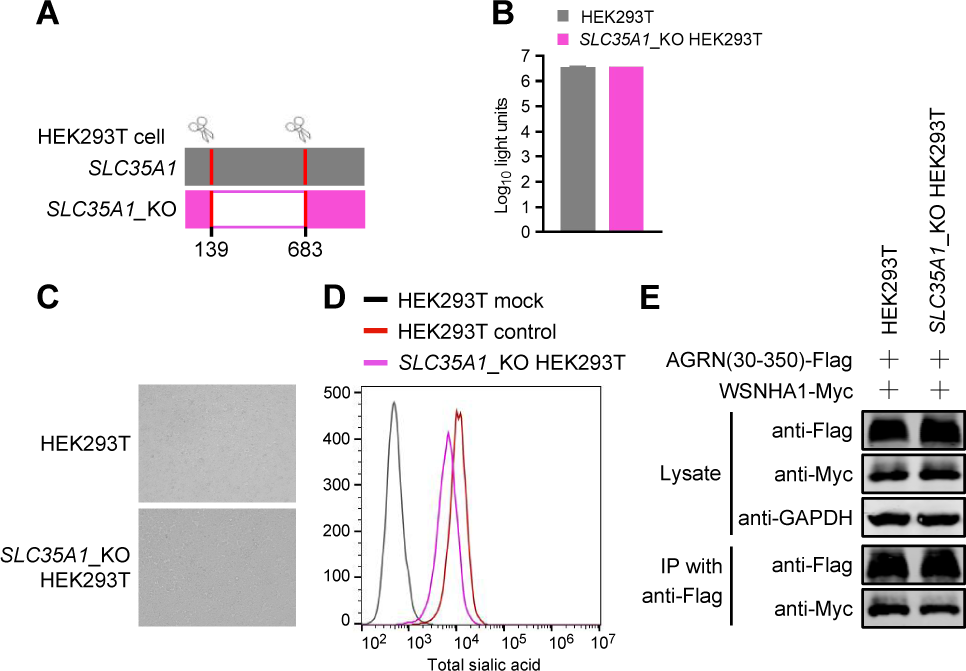


**Figure S9 The interaction between IAV HA1 and the key domain of AGRN is independent of sialic acids.** (A) Schematic diagram of targeting sites of a pair of gRNAs at the *SLC35A1* gene loci and the corresponding truncated mutant validated by sequencing. (B) Viability of *SLC35A1*_KO HEK293T cells was analyzed by using a CellTiter-Glo assay (n = 3 biologically independent samples). (C) Cellular morphology of *SLC35A1*_KO HEK293T cells. (D) *SLC35A1*_KO or control HEK293T cells were stained with wheat germ agglutinin (WGA) and then analyzed by flow cytometry to detect total sialic acids. (E) Plasmids expressing WSNHA1-Myc and AGRN(30-350)-Flag were co-transfected into *SLC35A1*_KO or control HEK293T cells. At 36 hours post-transfection, cell lysates were immunoprecipitated with a mouse anti-Flag mAb, and then subjected to western blotting with the indicated antibodies.


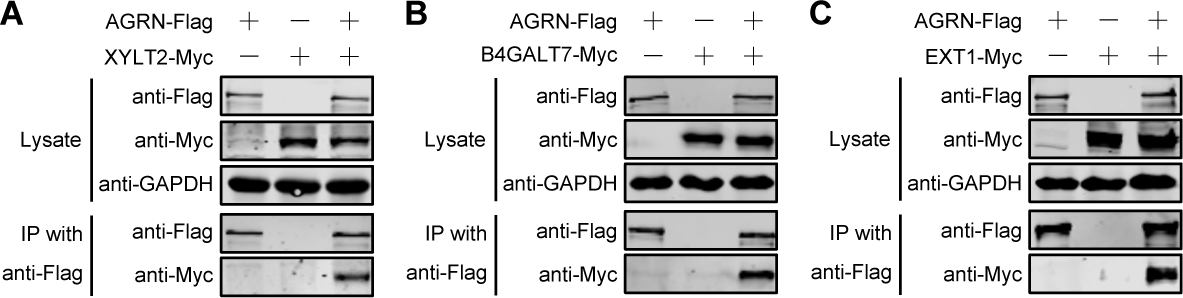


**Figure S10 AGRN interacts with** **the catalyzing molecules of HS synthesis.** (A to C) Plasmids expressing AGRN-Flag and XYLT2-Myc (A), B4GALT7-Myc (B), or EXT1-Myc (C) were transfected individually or co-transfected into HEK293T cells. At 36 hours post-transfection, cell lysates were immunoprecipitated with a mouse anti-Flag mAb, and then subjected to western blotting with the indicated antibodies.


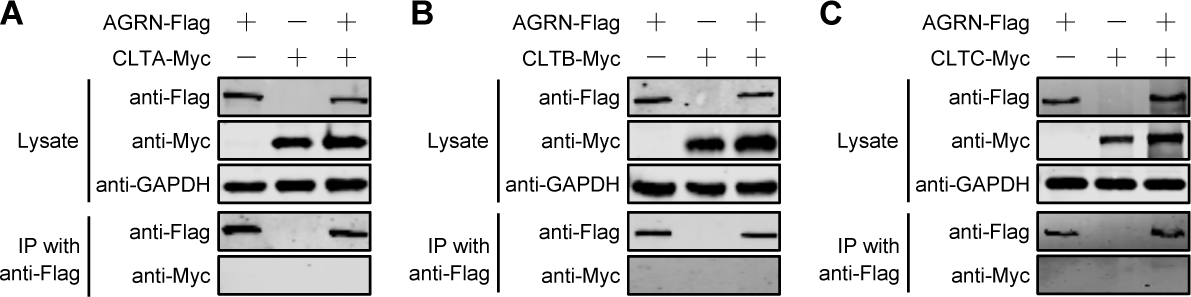


**Figure S11 AGRN does not interact with the subunits of clathrin (CLTA, CLTB and CLTC).** (A to C) Plasmids expressing AGRN-Flag and CLTA-Myc (A), CLTB-Myc (B), or CLTC-Myc (C) were transfected individually or co-transfected into HEK293T cells. At 36 hours post-transfection, cell lysates were immunoprecipitated with a mouse anti-Flag mAb, and then subjected to western blotting with the indicated antibodies.
